# Surface Molecularly Engineered Mitochondria Conduct Immunophenotype Repolarization of Tumor‐Associated Macrophages to Potentiate Cancer Immunotherapy

**DOI:** 10.1002/advs.202403044

**Published:** 2024-08-09

**Authors:** Cai‐Ju Zhang, Jia‐Mi Li, Dan Xu, Dan‐Dan Wang, Ming‐Hui Qi, Feng Chen, Bo Wu, Kai Deng, Shi‐Wen Huang

**Affiliations:** ^1^ Department of Radiology, Zhongnan Hospital of Wuhan University Wuhan University Wuhan 430071 China; ^2^ Department of Radiology Hainan Hospital Affiliated to Hainan Medical University Hainan 570311 China; ^3^ Key Laboratory of Biomedical Polymers of Ministry of Education, Department of Chemistry Wuhan University Wuhan 430072 China; ^4^ Department of Radiology Renmin Hospital of Wuhan University Jiefang Road 238,Wuchang District Wuhan Hubei 430060 China; ^5^ Department of Nuclear Medicine, Zhongnan Hospital of Wuhan University Wuhan University Wuhan 430071 China; ^6^ Department of Orthopedic Trauma and Microsurgery Zhongnan Hospital of Wuhan University Wuhan 430071 China

**Keywords:** immunotherapy, metabolic regulation, mitochondrial transplantation, surface molecular engineering, tumor‐associated macrophage

## Abstract

Reprogramming tumor‐associated macrophages (TAMs) to an inflammatory phenotype effectively increases the potential of immune checkpoint blockade (ICB) therapy. Artificial mitochondrial transplantation, an emerging and safe strategy, has made brilliant achievements in regulating the function of recipient cells in preclinic and clinic, but its performance in reprogramming the immunophenotype of TAMs has not been reported. Here, the metabolism of M2 TAMs is proposed resetting from oxidative phosphorylation (OXPHOS) to glycolysis for polarizing M1 TAMs through targeted transplantation of mannosylated mitochondria (mPEI/M1mt). Mitochondria isolated from M1 macrophages are coated with mannosylated polyethyleneimine (mPEI) through electrostatic interaction to form mPEI/M1mt, which can be targeted uptake by M2 macrophages expressed a high level of mannose receptors. Mechanistically, mPEI/M1mt accelerates phosphorylation of NF‐κB p65, MAPK p38 and JNK by glycolysis‐mediated elevation of intracellular ROS, thus prompting M1 macrophage polarization. In vivo, the transplantation of mPEI/M1mt excellently potentiates therapeutic effects of anti‐PD‐L1 by resetting an antitumor proinflammatory tumor microenvironment and stimulating CD8 and CD4 T cells dependent immune response. Altogether, this work provides a novel platform for improving cancer immunotherapy, meanwhile, broadens the scope of mitochondrial transplantation technology in clinics in the future.

## Graphical abstract

M1 mitochondria, extracted from inflammatory M1 macrophages, are engineered with mannosylated polyethyleneimine (mPEI/M1mt) to metabolically repolarize the immunophenotype of M2 tumor‐associated macrophages, acquiring an immune‐supportive tumor microenvironment (TME) and enhancing tumor anti‐PD‐L1 therapy. This approach not only expands the range of mitochondrial transplantation technology but also offers a new and promising clinical strategy for treating various types of tumors.

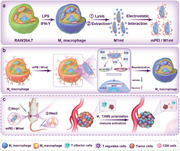

## 1 Introduction

Traditional cancer treatment strategies, such as surgical resection, chemotherapy, radiotherapy, etc. have made great progress in the treatment of solid tumors in clinics, but they still cannot effectively inhibit cancer recurrence and metastasis.^[^ ^1^, ^2^ ^]^ In recent years, immunotherapy has become a potent regimen that is expected to break through the bottleneck of traditional cancer treatment.^[^ ^3^, ^4^, ^5^, ^6^ ^]^ Generally, immunotherapy activates the host immune system by exploiting tumor vaccines, immune checkpoint inhibitors, and adoptive T‐cell therapy to monitor, identify, and attack cancer cells.^[^ ^7^, ^8^, ^9^, ^10^, ^11^ ^]^ Among these immunotherapy regimens, immune checkpoint blockade (ICB) therapies, which utilize FDA‐approved immune checkpoint inhibitors, such as programmed cell death protein 1/ligand 1 (PD‐1/PD‐L1) and cytotoxic T‐lymphocyte antigen 4 (CTLA‐4) for cancer therapy, have shown optimistic clinical responses in inhibiting both primary and metastatic tumors.^[^ ^12^, ^13^, ^14^ ^]^ It is worth noting, however, that less than 20% of patients currently respond to immune checkpoint therapy mostly due to immunosuppressive tumor microenvironment (TME).^[^ ^15^, ^16^, ^17^, ^18^ ^]^ The evidence has demonstrated that the infiltration and residence of tumor‐associated macrophages (TAMs) in TME is one of the main mechanisms of impairing ICB therapy, enabling TAMs to become promising targets for improving immunosuppressive TME to potentiate cancer immunotherapy.^[^ ^19^, ^20^ ^]^ TAMs are highly plastic and can be categorized into tumoricidal M1‐type and tumor‐supportive M2‐type macrophages according to their immunophenotype in TME.^[^ ^21^ ^]^ M1 TAMs can reverse the immunosuppressive TME and kill tumor cells by presenting antigens, secreting pro‐inflammatory factors (TNF‐α, IL‐6, IFN‐γ, etc.), and releasing reactive oxygen species (ROS, eg. H_2_O_2_, ClO^−^), etc. On the contrary, M2 macrophages promote tumor growth by secreting immunosuppressive factors (TGF‐β, IL‐4, IL‐10, etc.) and mediating angiogenesis, metastasis, and invasion. It has been reported that the vast majority of TAMs in tumor tissues are M2 TAMs due to the infiltrating and resident macrophages tend to polarize to tumor‐supportive type macrophage under stimulated with interferon regulatory factors, transcription factors, hypoxia, etc. in TME.^[^ ^23^, ^24^, ^25^, ^26^, ^27^, ^28^ ^]^ Therefore, the repolarization of M2 TAMs to M1 TAMs in TME has great potential for enhancing cancer ICB therapy.

The metabolic pattern of macrophages is related to the transformation and maintenance of their immunophenotype.^[^ ^29^ ^]^ In M1 TAMs, glycolysis is the dominant mode of energy metabolism, which induces the accumulation of citrate, succinate, and itaconate controlling TAMs toward an inflammatory phenotype.^[^ ^30^ ^]^ In the cytoplasm, citrate released from mitochondria mediates fatty acid synthesis, arginine metabolism, and redox homeostasis in macrophages. The upregulation of succinate activates the transcription of proinflammatory genes through stabilizing hypoxia‐inducible factor‐1α (HIF‐1α). In addition, the accumulation of itaconate damages the electron transport chain (ETC) and induces the generation of mitochondrial ROS (mROS), contributing to an inflammatory phenotype of macrophages. Inversely, M2 TAMs obtain energy through an integrally oxidative TCA cycle coupled to oxidative phosphorylation (OXPHOS), exhibiting reduced glycolysis and vigorously anti‐inflammatory phenotype. In M2 TAMs, mitochondrial respiration, accelerated by the high level of isocitrate dehydrogenase (IDH) and succinate dehydrogenase (SDH), inhibits the generation of inflammatory mediators, and the transcription of inflammatory genes. Meanwhile, the enhanced OXPHOS accelerates the ETC reaction on the mitochondrial membrane, reducing the production of inflammatory mediators in M2 macrophages. Thus, metabolic reprogramming is expected to become a promising strategy for repolarizing M2 TAMs to improve immunosuppressive TME.

Recent studies revealed that mitochondria are mainly responsible for the metabolic activities of cells.^[^ ^31^, ^32^ ^]^ Mitochondria serve as part of a feedback loop, communicating with other organelles to regulate cell metabolism by releasing signaling molecules, including ROS, Ca^2+^, NO, and other metabolites. In addition to regulating the metabolic activities of autologous cells, mitochondria can mediate the metabolism of recipient cells by transferring from autologous cells to adjacent recipient ones.^[^ ^33^ ^]^ The intercellular mitochondria transformation is a universal phenomenon in both physiological and pathological environments, improving the vitality and function of recipient cells. In this regard, allogeneic or autologous mitochondrial transplantation treatment strategies have been boldly proposed. This strategy involves transferring healthy mitochondria isolated and purified from normal tissues and cells to target tissues with dysfunctional mitochondria.^[^ ^34^ ^]^ For example, the transplantation of mouse astrocyte‐derived mitochondria shows neuroprotective effects against ischemic stroke by enhancing the metabolism to decrease apoptosis‐related gene expression of neurons.^[^ ^35^ ^]^ The transplantation of mitochondria isolated from nontumorigenic breast epithelial cells (MCF‐12A) increases the chemosensitivity of MCF‐7 by improving the OXPHOS ratio to activate apoptotic signal activation and transmission.^[^ ^36^ ^]^ In addition, considerable efforts have been devoted to progressing mitochondrial transplantation for patients. In a clinical trial, the healthy mitochondria from the parent are transferred to CD34^+^ hematopoietic stem cells of the patient, and then the expanded hematopoietic stem cells are transplanted back to the patient for the treatment of single large‐scale mitochondrial DNA deletion syndromes.^[^ ^37^ ^]^ Compared with traditional drugs, the transplantation of active mitochondria exhibits more advantages in cell bioenergetic transformation. On the one hand, mitochondria have no dose‐limiting toxicity, broadening their way for clinical application. On the other hand, the therapeutic property of mitochondrial transplantation is multifaceted as it can mediate genetic information transformation, protein synthesis, and metabolite secretion, simply but powerfully regulating cell metabolism. Furthermore, mitochondria, possessing circular and double‐stranded DNA molecules, are self‐replicating and self‐autonomous in recipient cells.^[^ ^38^ ^]^ Therefore, transplanting mitochondria into M2 TAMs has significant advantages and clinical application prospects in polarizing M1 TAMs and ameliorating immunosuppressive TME, but it has not been reported so far.

**Scheme 1:**
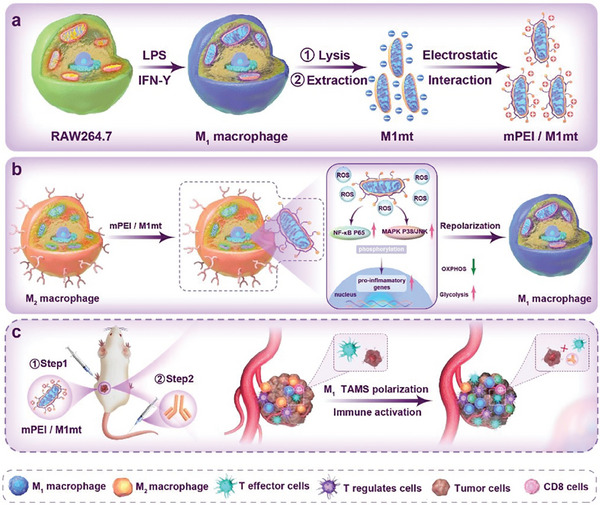
Schematic illustration of engineered mitochondria reprogramming M2 TAMs for generating inflammatory TME and potentiating cancer ICB therapy. a) Mitochondria isolated from M1 macrophages are surface molecularly engineered with mPEI to form mPEI/M1mt. b) The mechanism of mPEI/M1mt in M2 macrophage reprogramming: mPEI/M1mt accelerates the phosphorylation of NF‐κB p65, MAPK p38, and JNK by glycolysis‐mediated elevation of intracellular ROS. c) The combination of mPEI/M1mt and anti‐PD‐L1 efficiently reprograms TEM and potentiates cancer immunotherapy.

Herein, we proposed selective transplantation of active mitochondria isolated from M1 macrophages to reprogram the immunophenotype of M2 TAMs for generating inflammatory TME and potentiating ICB cancer therapy (**Scheme** **1**). The M2 macrophages targeted M1mt (mPEI/M1mt) were prepared from the electrostatic interaction between negatively charged M1mt and positively charged mannosylated PEI_1800_ (mPEI). We found that mPEI/M1mt with uniform nanoscale could positively accumulate into M2 macrophages compared with uncoated free M1mt. After transplantation, mPEI/M1mt exhibited excellent biocompatibility and effectively repolarized M2 macrophages to a potent pro‐inflammatory and cytotoxic M1 phenotype by regulating metabolism from OXPHOS to glycolysis. Mechanistically, mPEI/M1mt accelerated the phosphorylation of NF‐κB p65, MAPK p38, and JNK by glycolysis‐mediated elevation of intracellular ROS. After intratumor injection, mPEI/M1mt not only exhibited robust antitumor efficiency as monotherapy but also potentiated anti‐PD‐L1 therapy in two tumor models. In contrast, anti‐PD‐L1 elicited dispirited antitumor efficiency as monotherapy. Immunological analysis showed that mPEI/M1mt programmed an antitumor TME that was the elevation of the percentage of pro‐inflammatory M1 TAMs and activation of CD8^+^ and CD4^+^ T cells‐mediated adaptive immune response.

## 2 Results and Discussion

### 2.1 Construction and Characterization of Mannosylated Mitochondria

The surface modification of mitochondria with biomaterials is significant and feasible for mitochondrial transplantation.^[^ ^32^, ^39^, ^40^ ^]^ Previous studies have revealed that mitochondria functionalized with biocompatible polymers endow the ability to efficiently enter the recipient cells, improving the efficiency of mitochondrial transplantation. The free mitochondria can be decorated with positively charged materials, such as polyethyleneimine (PEI), due to their membrane possessing considerable negative charge (−20 to −180 mV). Additionally, mannose receptors are over‐expressed on the M2‐type macrophage membrane, which can accelerate the uptake of mannosylated biological materials. Therefore, M2 macrophage targeted and positively charged polymers mannosylated PEI (Mn = 1800) (mPEI) were designed to modify the mitochondrial surface through electrostatic interaction. As depicted in Figure S1 (Supporting Information), mPEI were fabricated by the sequential conjunction of hydrazine with PEI_1800_‐methyl acrylate and mannose. The chemical structure of intermediates and the final product mPEI were analyzed and confirmed with ^1^H NMR spectroscopy (Figure S2, Supporting Information).

To collect active mitochondria in M1 and M2 macrophages, we first separately polarize the mouse macrophages RAW 264.7 (M0 macrophage) with an inflammatory cocktail interferon‐γ (IFN‐γ) (300 ng mL^−1^) and lipopolysaccharides (LPS) (100 ng mL^−1^), and anti‐inflammatory cytokine IL‐4 (10 ng mL^−1^). The efficiency of M1 and M2 macrophage programming was determined by detecting the expression of CD86 (M1 marker) and CD206 (M2 marker) through flow cytometry (FCM). As shown in Figure S3 (Supporting Information), RAW 264.7 cotreated with IFN‐γ and LPS exhibited a higher expression of CD86 than untreated RAW 264.7 and IL‐4 stimulated RAW 264.7. In contrast, RAW 264.7 stimulated with IL‐4 showed a higher expression of CD206 signal than RAW 264.7, and the inflammatory cocktail treated RAW 264.7. These results revealed the achievement of programming of M1 and M2 macrophages. Then, the polarized macrophages were stained with a Mito‐Tracker Deep Red probe to label the intracellular mitochondria before being isolated with a standard protocol. In Figure S4 (Supporting Information), the plenty of uneven red aggregated pieces in suspension demonstrated the successful sieving of active mitochondria (M1mt or M2mt). The isolated mitochondria were immediately stored at ‐80 °C in stock solution for further experiment.

**Figure 1:**
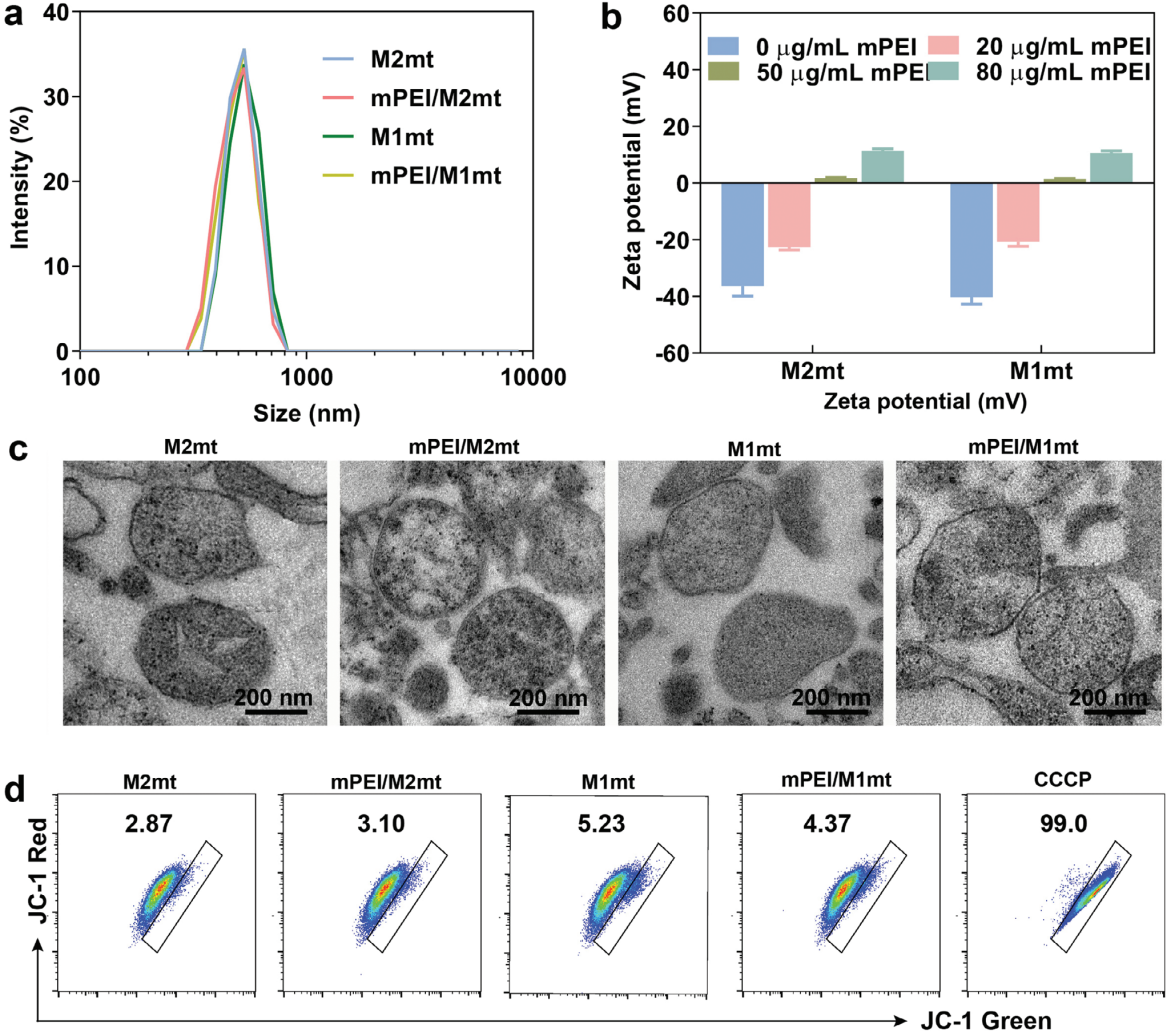
Characterization of mitochondria before and after mPEI modification. a) DLS analyzed the size distribution of mitochondria before and after mPEI decoration. b) Zeta potential of mitochondria treated without or with different concentrations of mPEI. c) TEM images of M2mt, mPEI/M2mt, M1mt, mPEI/M1mt. (Scale bar = 200 µm). d) Flow cytometry analysis of the fluorescence intensity of JC‐1 green and red to determine the integrity of the mitochondria membrane.

The average size of M1mt and M2mt was characterized to be 513 nm and 496 nm by dynamic light scattering (DLS) analysis (**Figure** **1**a). And the extracted M1mt and M2mt have negatively charged surface potential (−40.2 mV and −36.3 mV), which may hinder the M2 macrophages endocytosis due to the electrostatic repulsion between mitochondria and macrophage membrane (Figure 1b). For endowing mitochondria to target M2 macrophages, mPEI was separately dissolved in a solution containing isolated M1mt and M2mt, forming mPEI/M1mt and mPEI/M2mt. The surface potential of M1mt and M2mt was regulated from a negative charge to a positive charge with the addition of mPEI, indicating successful decorating of the mitochondria (Figure 1b). Considering that mitochondria with a positive charge may accelerate cellular internalization, mannosylated mitochondria prepared from mitochondria and mPEI at a concentration of 5 × 10^6^ mitochondria/mL and 80 µg mL^−1^, respectively, for further experiment. As shown in Figure 1a, mitochondrial surface modification with mPEI has negligible changes in their size distribution, and the average size of mPEI/M1mt and mPEI/M2mt is nearly the same as free mitochondria M1mt and M2mt. The morphologies of the free and mannosylated mitochondria in a dried state, observed with TEM, were nearly sphere‐shaped (Figure 1c). The activity of free and mannosylated mitochondria was detected by JC‐1. JC‐1 is a positively charged fluorescent dye that exhibits potential‐dependent accumulation in mitochondria, monitoring the health of mitochondria. JC‐1 forms J‐aggregates on healthy mitochondrial membranes and emits red fluorescence (590 nm), while it exists in the form of monomers on damaged mitochondrial membranes and emits green fluorescence (529 nm). The free and mannosylated mitochondria exhibited a high level of red fluorescence signal and negligible green fluorescence signal (Figure 1d), while mPEI/M1mt treated with a mitochondrial membrane disruption agent carbonyl cyanide m‐chlorophenyl hydrazine (CCCP) showed high green fluorescence signal, revealing the integrity of the free and mannosylated mitochondrial membrane. Therefore, mPEI/M1mt and mPEI/M2mt, characterized with intact membrane structures and nanoscale, could accommodate them to biomedicine in vitro and in vivo.

### 2.2 Mannosylated Mitochondria Facilitated M2 Macrophage Endocytosis and Reprogramming

**Figure 2:**
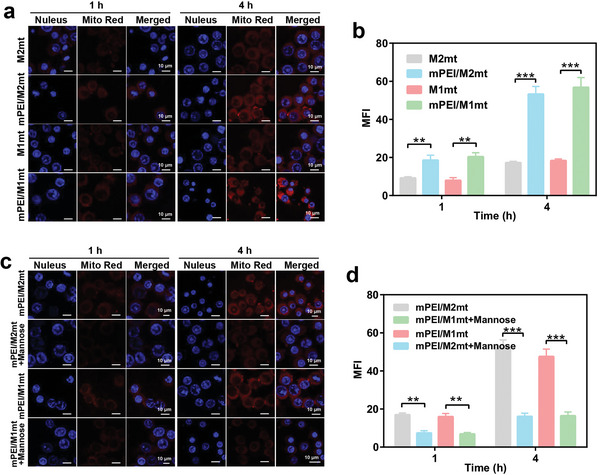
The internalization of M2mt, mPEI/M2mt, M1mt, mPEI/M1mt by M2 macrophages was observed by CLSM. a, b) CLSM images and quantification of the internalization of M2mt, mPEI/M2mt, M1mt, mPEI/M1mt (Mito Tracker Deep Red labeling) by M2 cells after incubated for 1 h and 4 h. c, d) CLSM images and quantification of the internalization of mPEI/M2mt and mPEI/M1mt by M2 macrophages pretreated with or without mannose. Mean ± SD, n = 3, **P<0.01; ***P<0.001.

Polymer‐modified mitochondria with a neutral and positive charge exhibited better cellular internalization compared with free mitochondria.^[^ ^39^, ^40^, ^41^ ^]^ On this basis, we speculated that mannosylated mitochondria could further accelerate M2 macrophage endocytosis due to the high expression of mannose receptors on the membrane of M2 macrophage. To determine cellular internalization, the Mito‐Tracker Deep Red probe labeled free and mannosylated mitochondria incubated with M2 macrophage before observing with CLSM and quantitively analyzing with FCM, respectively. The Mito‐Tracker Deep Red probe labeled mitochondria exhibited red fluorescence in CLSM images. The fluorescence images revealed that the cellular internalization of mannosylated mitochondria by M2 macrophages was in a time‐dependent manner, which was proved by the increase of red fluorescence with incubation time (**Figure** **2**a). The cellular internalization of mPEI/M1mt and mPEI/M2mt occurred as early as 1 h after incubation with M2 macrophages, and exhibited intensive internalization at a time point of 4 h. However, M2 macrophages had a passive performance in the uptake of free mitochondria, exhibiting weak red fluorescence even at the incubation time of 4 h. Quantitative analysis by Image J software revealed that the red fluorescence intensity in mPEI/M1mt treated M2 macrophages was 2.9‐fold higher than that in free M1mt treated M2 macrophages at the incubation time of 4 h (Figure 2b). The result of FCM analysis showed a similar trend (Figure S5, Supporting Information). Next, mannosylated mitochondria uptake by M2 macrophages was evaluated in the presence of mannose, which can down‐regulate the cellular uptake mediated by mannose receptors. M2 macrophages pretreated with mannose dramatically inhibited the cellular internalization of mannosylated mitochondria (Figure 2c,d; Figure S6, Supporting Information). In mPEI/M1mt group, the red fluorescence intensity in mannose pre‐treated M2 macrophages was 3.1‐fold lower than that in M2 macrophages without mannose pre‐treated at the incubation time point of 4 h. Meanwhile, after M2 macrophages were pre‐treated with mannose, the red fluorescence intensity in mPEI/M1mt group was equivalent to that in the free M1mt group. These results demonstrated the efficiency of mitochondrial transplantation into M2 macrophages.

Once the recipient cell absorbs free mitochondria, they are carried to the endosome and lysosome. The ability of mitochondria to escape from lysosomes may significantly impact their function in repolarizing M2 macrophages. Therefore, the intracellular localization of M1mt and mPEI/M1mt in M2 macrophage was investigated by confocal laser scanning microscopy (CLSM). After incubation for 3 h, the merged images in Figure S7 (Supporting Information) depicted an obvious yellow color, indicating that most of the M1mt and mPEI/M1mt were captured by lysosomes. In merged images, the color changed from yellow to red when M1mt and mPEI/M1mt escaped from lysosomes due to the poor overlap of the lysosomal tracker with the MitoTracker. With the extension of incubation time, the reinforced red color of merged images was observed in mPEI/M1mt treated M2 macrophages, implying that mPEI/M1mt exhibited significant endosomal escape. While free mitochondria M1mt showed low lysosomal escape even when incubated for 12 hours. The conspicuous endosomal escape ability of mPEI/M1mt may be due to the “proton‐sponge” effect of mannosylated polyethyleneimine (mPEI) decorated on the surface of mitochondria.^[^ ^41^ ^]^ These results demonstrated that mitochondria decorated with mannosylated polyethyleneimine (mPEI) exhibited high performance in lysosomal escape, endowing them with the ability to move to the cytoplasm and repolarize M2 macrophages.

The cytotoxicity of mannosylated mitochondria is as important as that of endocytosis for mitochondrial transplantation because high cytotoxicity of the graft would hinder the efficiency of M2 macrophage reprogramming. The cytotoxicity of mPEI, free mitochondria, and mannosylated mitochondria was first determined in M2 macrophages and 4T1 cancer cells by the MTT method. As shown in Figure S8 (Supporting Information), although a high concentration of macromolecule PEI has been reported to have certain toxicity to cells, mPEI was innocuous to M2 macrophages and 4T1 cancer cells within the test concentration range. Excitedly, free mitochondria and mannosylated mitochondria were also biocompatible with M2 macrophages and 4T1 cancer cells. In addition, after incubation with mPEI, free mitochondria, and mannosylated mitochondria, the strong red fluorescence intensity of JC‐1 in M2 macrophages demonstrated the integrity of the mitochondrial membrane (Figure S9, Supporting Information). Hence, based on the wonderful cell internalization and viability, it could be inferred that mPEI/M1mt would greatly mediate M2 macrophage repolarization.

**Figure 3:**
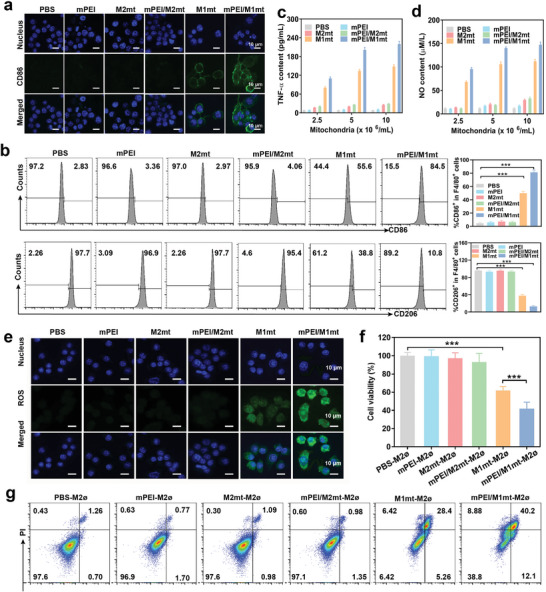
M2 macrophage reprogramming. a) The CD86 expression in M2 macrophages after mitochondrial transplantation was observed by CLSM. b) Flow cytometry (FCM) analysis of CD86 and CD206 in M2 macrophages after mitochondrial transplantation. The production of c) TNF‐α and d) NO in the supernatant of M2 macrophages that incubated with different concentrations of mPEI, M2mt, mPEI/M2mt, M1mt, and mPEI/M1mt for 24 h. e) The intracellular ROS of M2 cells after treatment with mPEI, M2mt, mPEI/M2mt, M1mt, and mPEI/M1mt was observed by CLSM. f, g) Cell viability of 4T1 cells after treatment with M2 macrophages that were treated with mPEI, M2mt, mPEI/M2mt, M1mt, and mPEI/M1mt were separately determined by (f) MTT methods and (g) FCM. Mean ± SD, n = 3, **P<0.01; ***P<0.001.

We next evaluated the potential of mitochondrial transplantation to mediate M2 macrophage reprogramming in vitro. Pro‐inflammation M1 macrophages and anti‐inflammation M2 macrophages exhibited different morphologies (Figure S10, Supporting Information). M2 macrophages display a round and flattened cellular shape, whereas M1 macrophages exhibit an elongated fusiform morphology. Following incubation with mPEI/M1mt for 24 h, the M2 macrophages underwent a morphology change resembling that of M1 macrophages. The classical pro‐inflammatory M1 macrophages are characterized by the expression of costimulatory molecules and the secretion of inflammatory cytokines and mediators. We first detected the expression of CD86, CD80, and CD206 in free and mannosylated mitochondria‐treated M2 macrophages. After transplantation of mPEI/M1mt, M2 macrophages increased the expression of costimulatory molecule CD86 on the membrane, which was validated by the high green fluorescence of PE‐anti‐CD86 in CLSM images (**Figure** **3**a). The moderate upregulation of CD86 in free M1mt treated M2 macrophages due to low cellular internalization. As a control, the other three groups including mPEI, M2mt, mPEI /M2mt exhibited no positive impact on M1 macrophage marker expression. Quantitative analysis with FCM further demonstrated that M2 macrophages treated with mPEI/M1mt significant increase in the expression of CD86 (84.5%) and CD80 (87.7%) and a concomitant decrease in the expression of CD206 (10.8%) (Figure 3b; Figure S11, Supporting Information). Meanwhile, a high concentration of mPEI/M1mt induced more CD86 expression, which revealed the expression of CD86 was concentration‐dependent on M1mt (Figure S12, Supporting Information). Moreover, M1 macrophages can mediate tumor cell necrosis or apoptosis and induce antitumor immune response by secretion of inflammatory cytokines and mediators, including tumor necrosis factor‐α (TNF‐α), NO, Interferon‐γ (IFN‐γ), Interleukin‐1 (IL‐1) and ROS. As depicted in Figure 3c,d and Figures S13,S14 (Supporting Information), M2 macrophages transplanted with mPEI/M1mt secreted a large amount of TNF‐α and NO, which also coincided with the concentration of mPEI/M1mt. Free M1mt treated M2 macrophages also induced upregulation of TNF‐α and NO to a lower level than dose‐matched mPEI/M1mt. Similarly, the production of IFN‐γ and IL‐1 remarkably enhanced in M2 macrophages treated with mPEI/M1mt (5 × 10^6^/mL) for 24 h, Figure S15a, b (Supporting Information). Furthermore, the generation of ROS was imaged and detected by DCF green fluorescence, which was generated by the oxidation of nonfluorescent DCFH under ROS stimulation (Figure 3e). M2 macrophages treated with mPEI/M1mt exhibited strong green DCF fluorescence, indicating the upregulation of intracellular ROS. Consistent with inflammatory cytokines, M2 macrophages treated with free M1mt generated lower levels of ROS than those treated with mPEI/M1mt. M2 macrophages treated with PBS, free M2mt, and mPEI/M2mt undoubtedly generated negligible ROS. To further confirm mPEI/M1mt could reprogram protumoral M2 macrophages to tumoricidal M1 macrophages, the anti‐inflammatory cytokines IL‐10 and TGFβ were measured. In Figure S15c, d (Supporting Information), M2 macrophages treated with mPEI/M1mt decreased the secretion of anti‐inflammatory cytokines IL‐10 and TGFβ. Free M1mt treated M2 macrophages also induced a decline of IL‐10 and TGFβ to a lower level than dose‐matched mPEI/M1mt. PBS, free M2mt, and mPEI/M2mt treated M2 macrophages undoubtedly exhibited negligible changes in the secretion of IL‐10 and TGF‐β. Intriguingly, transplantation of M1mt into M2 macrophages could inhibit 4T1 cell viability to a large extent, and mPEI/M1mt treated M2 macrophages were prominent in inducing 4T1 cell death that may be due to the secretion of the high‐level of inflammatory cytokines, including TNF‐α, NO, IFN‐γ, IL‐1, and ROS. These data revealed that the transplantation of mPEI/M1mt could efficiently reprogram protumoral M2 macrophages to tumoricidal M1 macrophages in vitro, exhibiting prominent potential in reinforcing tumor immunotherapy.

### 2.3 The Mechanism of mPEI/M1mt Reprogrammed M2 Macrophages

**Figure 4:**
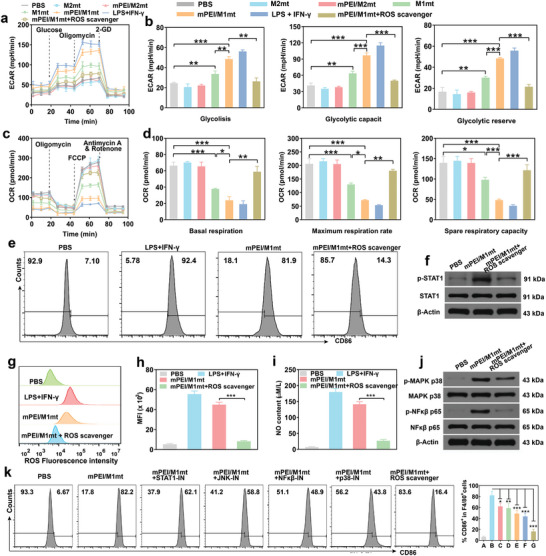
The mechanism of mitochondrial transplantation mediated M1 macrophage polarization. a, c) Glycolysis and mitochondrial stress tests were performed to determine the metabolism of M2 macrophages after different treatments using a Seahorse XF96 Extracellular Flux assay kit. b) Glycolysis, glycolysis capacity, and glycolysis reserve of M2 macrophage after transplanted with different types of mitochondria were obtained according to the representative extracellular acidification rates (ECAR). d) Basal respiration, maximum respiration rate, and spare respiration capacity were obtained according to the oxidative phosphorylation oxygen consumption rate (OCR). The role of ROS in mPEI/M1mt transplantation mediated M2 macrophage polarization. e) FCM analyzed the expression of CD86 and f) Western blotting (WB) analysis of the expression of p‐STAT1 in M2 macrophages treated with mPEI/M1mt in the presence and absence of ROS‐scavenger N‐acetylcysteine. g, h) FCM analysis of ROS generation in M2 macrophage transplanted with mPEI/M1mt in the presence and absence of ROS‐scavenger N‐acetylcysteine. i) The content of NO in the supernatant of M2 macrophage culture medium after different treatments. j) ROS mediated the phosphorylation of NF‐κB p65 and MAPK p38 in M2 macrophages transplanted with mPEI/M1mt in the presence and absence of ROS‐scavenger N‐acetylcysteine. k) FCM analysis and corresponding statistical data of the expression of CD86 in M2 macrophages after being treated with mPEI/M1mt and different inhibitors. STAT1‐IN: inhibitor of STAT1 (Fludarabine); JNK‐IN: inhibitor of JNK (JNK‐IN‐8); NFkβ‐IN: inhibitor of NFkβ (JSH‐23); p38‐IN: inhibitor of p‐MAPK p38 (p38 MAPK‐IN‐1). A‐G represent: PBS, NFkβ‐IN, mPEI/M1mt+ STAT1‐IN, mPEI/M1mt+JNK‐IN, mPEI/M1mt+ NFkβ‐IN, NFkβ‐IN+ROS scavenger. Mean±SD, n = 3, *P < 0.05; **P<0.01; ***P<0.001.

Previous reports have declared that mitochondrial transplantation can reset cell metabolism to restore or reprogram the phenotype and function of recipient cells.^[^ ^42^, ^43^ ^]^ However, whether the transplantation of mitochondria isolated from M1 macrophages can regulate the metabolism of M2 macrophages has not been reported. Here, we decided to explore the effect of transplantation of mPEI/M1mt on M2 macrophage metabolism through Seahorse assay. Generally, M1 macrophages tend to glycolysis, while M2 macrophages rely on oxidative phosphorylation (OXPHOS). A low basal extracellular acidification rate (ECAR) was detected in free and mannosylated M2mt treated M2 macrophages, while an M1 macrophage metabolic type enhanced ECAR was observed in free and mannosylated M1mt treated M2 macrophages. It was noteworthy that mPEI/M1mt treatment induced the highest level of ECAR in M2 macrophages, which was further potentiated by the sequential addition of glucose and oligomycin (**Figure** **4**a). The high level of ECAR demonstrated that mPEI/M1mt could accelerate M2 macrophage glycolysis, which coincided with the increase of glycolytic capacity and glycolytic reserve (Figure 4b). To explore the mitochondrial OXPHOS in mPEI/M1mt treated M2 macrophages, oxygen consumption rate (OCR) was measured by sequentially adding oligomycin, carbonyl cyanide p‐trifluoromethoxyphenylhydrazone, and rotenone (Figure 4c). The low level of basal and max OCR indicated mPEI/M1mt treated M2 macrophages tend to an oxygen consumption pattern of M1 macrophages, namely anaerobic glycolysis, which was consistent with the low level of maximal respiration rate and spare respiratory capacity (Figure 4d). However, just as PBS, M2mt, and mPEI/M2mt had no effect on the regulation of the immunophenotype of M2 macrophages, their impact on the regulation of the metabolic type of M2 macrophages was also negligible. Above all, these data confirmed that transplantation of mPEI/M1mt effectively reset the metabolic pattern of M2 macrophages from anti‐inflammatory OXPHOS to pro‐inflammatory glycolysis.

To immersively understand the role of mPEI/M1mt‐mediated metabolic alternation in the polarization of M1 macrophages, we explored which metabolite participated in reprogramming the immunophenotype of M2 macrophages. ROS is an important signal transduction molecule, which is of great significance in regulating physiological and pathological processes, and has been reported to be involved in M2 macrophage reprogramming. Meanwhile, once glycolysis dominates the energy metabolism of macrophages, it elevates the level of intracellular ROS by inducing mitochondrial ETC damage and OXPHOS blockage. Thus, we paid attention to the molecular mechanism of ROS in metabolic alternation‐induced M2 repolarization. As depicted in Figure 4a–d, the metabolic type of mPEI/M1mt treated M2 macrophages was shifted from glycolysis to OXPHOS after eliminating intracellular ROS. By detecting the expression of M1 macrophages marker CD86 in mPEI/M1mt treated M2 macrophages (Figure 4e), we found that it was down‐regulated after diminished intracellular ROS through the addition of ROS‐scavenger N‐acetylcysteine, which was consistent with abolished phosphorylation of JAK STAT1, an inflammatory transcription factor (Figure 4f). The depletion of ROS (Figure 4g, h) also induced a reduction of NO generation in mPEI/M1mt treated M2 macrophages (Figure 4i). Collectively, these results revealed that ROS involved in mPEI/M1mt‐mediated M2 macrophages reprogramming. Furthermore, ROS has been reported to induce the accelerated transcription of inflammatory genes by activating the downstream signal of NF‐κB, mitogen‐activated protein kinase (MAPK) accelerating M1 polarization. Here, the phosphorylation of NF‐κB p65, MAPK p38 and JNK was detected in M2 macrophages treated with mPEI/M1mt, however, it was inhibited after the addition of N‐Acetylcysteine (Figure 4j; Figure S16, Supporting Information). Furthermore, the molecular mechanism of ROS‐mediated repolarization of M2 macrophages was confirmed by the addition of corresponding inhibitors (Figure 4k). Following the respective addition of the inhibitor of STAT1 (Fludarabine), JNK (JNK‐IN‐8), NFkβ (JSH‐23) and p‐MAPK p38 (p38 MAPK‐IN‐1), M2 macrophages treated with mPEI/M1mt showed reduced repolarization towards M1 macrophages by 62.1%, 58.8%, 48.9%, and 43.8%. Significantly, the incubation of ROS‐scavenger N‐acetylcysteine conspicuously reduced the repolarization rate of M2 macrophages treated with mPEI/M1mt to 16.4%, which was 3.7, 3.5, 2.9, and 2.6‐fold lower than that in the addition of Fludarabine, JNK‐IN‐8, JSH‐23, and p38 MAPK‐IN‐1, respectively. Therefore, we concluded that NF‐κB p65, MAPK p38 and JNK activated by ROS were required for mPEI/M1mt mediated M2 macrophage reprogramming.

### 2.4 mPEI/M1mt Potentiated the Anticancer Efficiency of Anti‐PD‐L1

**Figure 5:**
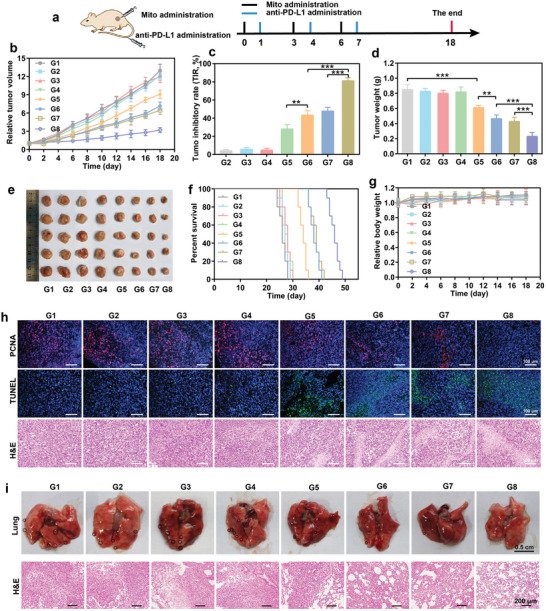
Antitumor effect of 4T1 tumor‐bearing mice model in vivo. a) Schematic diagram of mouse administration procedures. b) Tumor growth curves of tumor‐bearing mice under different treatments. c) Tumor inhibitory rate (TIR) of tumor growth. d) Weight of the tumors harvested on day 18 after different treatments. e) Digital images of tumors collected from mice on day 18 after different treatments. f) Survival curves of tumor‐bearing mice under different treatments. g) The curves of body weight changed over time in tumor‐bearing mice under different treatments. h) Immunofluorescence and H&E staining of tumor sections after different treatments. i) Images of tumor metastasis to the lung following different treatments. G1:PBS, G2:mPEI, G3:anti‐PD‐L1, G4:mPEI/M2mt+anti‐PD‐L1, G5:M1mt, G6:M1mt+anti‐PD‐L1, G7:mPEI/M1mt, G8:mPEI/M1mt+anti‐PD‐L1. Mean ± SD (n = 5). *P < 0.05, **P < 0.01, ***P < 0.001.

To investigate whether transplantation of M1mt could enhance the anticancer efficiency of anti‐PD‐L1 in vivo, we first established the subcutaneous 4T1 tumor‐bearing mice. 4T1 tumor, characterized by negative estrogen receptor (ER), progesterone receptor (PR), and human epidermal growth factor receptor‐2 (Her‐2), is a standard model for investigating the treatment of triple‐negative breast cancer (TNBC) in preclinic and clinic. Meanwhile, this model is ineffective in immune checkpoint blockade (ICB) therapies, including anti‐PD‐L1, due to the abundant and diverse bone marrow‐derived immunosuppressive cells in tumor tissues. After the tumor volume reached approximately 50 mm^3^, we first determined the dose of mitochondria for tumor therapy. The dose of mitochondria administered was determined by investigating the percentage of M2 TAMs reprogramming in tumors following intratumor injection of varying doses of mitochondria. Consistent with the in vitro results, the rate of M2 TAMs reprogramming was correlated with the content of mPEI/M1mt and reached a plateau at an injection content of 5×10^6^ mitochondria each time (Figure S17, Supporting Information). Therefore,, a single dose of 5×10^6^ mitochondria was administered in the next in vivo experiments. Next, 4T1‐tumor bearing mice were randomly divided into 8 groups (n = 5), including PBS, anti‐PD‐L1, mPEI + anti‐PD‐L1, mPEI/M2mt + anti‐PD‐L1, M1mt, M1mt + anti‐PD‐L1, mPEI/M1mt, mPEI/M1mt + anti‐PD‐L1, the detailed treatment scheme was shown in (**Figure** **5**a). The tumor size and mice weight were measured and recorded every two days (Figure S18, Supporting Information). Not surprisingly, the relative tumor volume (RTV) and tumor inhibition rate (TIR, %) revealed that a negligible effect on the inhibition of tumor growth was observed in the anti‐PD‐L1 alone group, the trends were consistent with the PBS, mPEI + anti‐PD‐L1, and mPEI/M2mt + anti‐PD‐L1 groups (Figure 5b, c). Compared to PBS, M1mt moderately inhibited tumor growth with RTV = 9.08 and TIR = 27.9%, however, it potentiated the anticancer therapy of anti‐PD‐L1 (RTV = 7.15, TIR = 43.3%). Interstingly, after being modified with mannosylated PEI, mPEI/M1mt exhibited higher anticancer efficiency (RTV = 6.45, TIR = 47.5%) in comparison with the combination of M1mt and anti‐PD‐L1. Typically, anti‐PD‐L1 combined with mPEI/M1mt remarkably delayed tumor growth (RTV = 3.17, TIR = 80.9%), exhibiting the lowest tumor weight and tumor size compared to other groups (Figure 5d, e). In combination with anti‐PD‐L1, mPEI/M1mt showed a better antitumor effect than free M1mt due to the mannosylated modification improved accumulation and retention of M1mt in M2 TAMs, which was indicated by the higher overlap of red (M1mt) and green fluorescence (M2 TAMs) (Figure S19, Supporting Information). Consistent with the trend of tumor inhibition, anti‐PD‐L1 alone could not improve the animal median survival time compared with PBS. Conversely, mPEI/M1mt combined with anti‐PD‐L1 immunotherapy significantly prolonged median survival time by 46 days compared with 26 days of median survival time of the PBS group (Figure 5f). The serum biochemical indices, including total bilirubin (TBil), alanine aminotransferase (ALT), aspartate aminotransferase (AST), γ‐glutamyl transpeptidase, serum creatinine (Scr) and serum urea nitrogen (SUN), exhibited no fluctuation after 7 days of treatments (Figure S20, Supporting Information). Meanwhile, all the mice exhibited negligible changes in body weight, and histological damage in major organ sections demonstrating the negligible systemical toxicity of this treatment strategy (Figure 5g; Figure S21, Supporting Information).

To further confirm the preferably strengthening effect of mPEI/M1mt in ICB therapy, terminal deoxynucleotidyl transferase dUTP nick end labeling (TUNEL) and proliferating cell nuclear antigen (PCNA) staining assay were applied to analyze the apoptosis and proliferation of tumor cells, respectively. All M1mt groups induced more apoptosis and stronger proliferation inhibition of tumor cells than other groups, including PBS, anti‐PD‐L1, mPEI + anti‐PD‐L1, mPEI/M2mt + anti‐PD‐L1 (Figure 5h). Combined with anti‐PD‐L1, the highest tumor inhibition of mPEI/M1mt was indicated by the weakest red proliferation signal in PCNA analysis and the strongest green apoptosis signal in TUNEL analysis. The potentiated antitumor efficiency of ICB therapy mediated by mPEI/Mamt was also revealed by histological analysis of hematoxylin and eosin (H&E) staining. The histological results of tumors treated with anti‐PD‐L1 alone showed dense tumor cells with intact structure. Pretreatment with M1mt, anti‐PD‐L1 exhibited a small number of tumor cell necrosis. Comparatively, mPEI/M1mt combination with anti‐PD‐L1 induced obvious tissue apoptosis characterized by most of the tumor cell separation and nuclear ablation compared to other groups (Figure 5h). Overcoming the metastasis of cancer cells from primary tumor sites to other organs or tissues is a great challenge for cancer therapy. Although ICB has been reported to be promising in inhibiting cancer cell metastasis, most cancers have no response due to immunosuppressive TME. Lung metastasis is one of the markers of breast cancer progression. As depicted in Figure 5i, anti‐PD‐L1 alone could not delay 4T1 cells metastasis indicated by a large number of nodes on the surface of the lung and proliferative cells in H&E staining. Fortunately, anti‐PD‐L1 combination with mPEI/M1mt excellently inhibited tumor metastasis demonstrated by negligible lung nodes and proliferative cells. Altogether, these data toughly indicated that mPEI/M1mt could significantly potentiate anti‐PD‐L1 cancer therapy for inhibiting tumor growth and metastasis.

### 2.5 mPEI/M1mt Ameliorated Immunosuppressive TME

**Figure 6:**
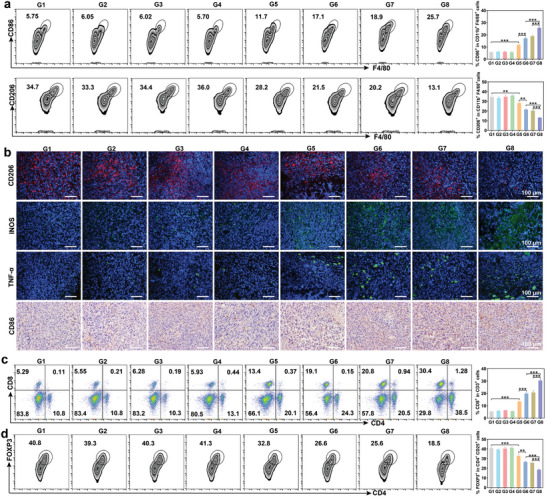
Evaluation of TAMs repolarization and immune cell regulation in vivo. a) The FCM analysis of M1 macrophages (CD86^+^ F4/80^+^ cells) and M2 macrophages (CD206^+^ F4/80^+^ cells) in the tumor tissue on day 7 after different treatments. Cells are gated by CD11b^+^ cells b) Immunofluorescence and immunohistochemical staining of M2 macrophages and M1 macrophages using different markers. c, d) The FCM analysis of (c) CD8^+^ T cells (gated by CD3^+^ T cells) and (d) Tregs (gated by CD25^+^ T cells) in tumor tissues after different treatments. G1:PBS, G2:mPEI, G3:anti‐PD‐L1, G4:mPEI/M2mt+anti‐PD‐L1, G5:M1mt, G6:M1mt+anti‐PD‐L1, G7:mPEI/M1mt, G8:mPEI/M1mt+anti‐PD‐L1. Mean±SD (n = 3), *P<0.05, **P<0.01, ***P<0.001.

To explore the mechanism of mPEI/M2mt potentiated anti‐PD‐L1 cancer therapy, the tumor tissues were first collected from sacrificed mice for immunological analysis after the last dosing for three days. M2 TAMs are the major class of immunosuppressive cells in TME, which dramatically limited ICB therapy by inhibiting the antitumor activity of T cells. Compared with the elimination of M2 TAMs, reprogramming them to M1 TAMs is more conducive to improving immunotherapy. To explore the repolarization of M2 TAM, tumor tissue single‐cell suspension was stained with M1 TAM (CD11b^+^F4/80^+^CD86^+^) and M2 TAM (CD11b^+^F4/80^+^CD206^+^) markers for FCM analysis (**Figure** **6**a). Compared with PBS, the low density of M1 TAM (6.02%) and a high population of M2 TAM (34.4%) in anti‐PD‐L1 treated tumors revealed that anti‐PD‐L1 alone was difficult to improve TEM. However, an increased number of M1 TAMs and a reduced population of M2 TAMs were measured in the transplantation of M1mt, indicating that M1mt could polarize M1 TAMs in vivo. Furthermore, in contrast to free M1mt (M1 TAM 11.7%), mPEI/M1mt induced more M1 TAMs (18.9%) polarization suggesting that targeting of TAMs was required for M1mt effectively reprogramming M2 TAMs. Although the effect of anti‐PD‐L1 on M2 TAM repolarization was negligible, the combination of anti‐PD‐L1 and mPEI/M1mt induced the most population of M1 TAM (25.7%), which was consistent with their superior antitumor efficiency. To directly visualize the impact of mPEI/M1mt on M1 TAM polarization, tumor tissues were sliced and stained for immunofluorescence and immunohistochemistry staining. The weaker fluorescence intensity of CD206, the stronger green fluorescence of iNOS and TNF‐α, and the upregulated expression of CD86 also indicated mPEI/M1mt had excellent performance in mediating M1 TAMs polarization, which was further enhanced in combination with anti‐PD‐L1(Figure 6b). These results demonstrated that the transplantation of mannosylated M1mt could effectively repolarize M2 TAMs to M1 TAMs in vivo.

The limitation of ICB therapy is that cytotoxic T cells at the tumor site are low‐infiltration and exhausted, and M2 TAMs repolarization is expected to reverse that situation. We first investigated the maturation of dendritic cells (DCs) (CD11c^+^CD80^+^CD86^+^) in lymph nodes as it can contribute to T cell‐based immune response, (Figure S22a, b, Supporting Information). Undoubtedly, the maturation rate of DCs in the mPEI/M1mt was significantly reinforced (22.8%), which was 1.9, 1.8, and 1.2‐fold higher than that of the PBS, mPEI, and M1mt treated group, respectively. Notably, the combination of mPEI/M1mt and anti‐PD‐L1 enhanced the maturation of DCs to the highest level (31.2%), exhibiting the potential to activate a strong T cell‐based immune response.

The immune activation was first determined by measuring the CD69^+^ T cells (CD3^+^CD8^+^CD69^+^) in lymph nodes (Figure S22c, d, Supporting Information). CD69, a disulfide‐linked homodimer protein, can be upregulated in most activated leukocytes, serving as an important maker of immune responses. Consistent with DCs maturation, the activation of CD69^+^ T cells in the combination of mPEI/M1mt and anti‐PD‐L1 has reached a plateau (14.9%), which was 3.8, 3.4, 2, 3.8, and 1.4‐fold higher than that of the PBS, mPEI, M1mt, anti‐PD‐L1, and M1mt + anti‐PD‐L1 treated group, respectively.

Myeloid‐derived suppressor cells (MDSCs) are a heterogeneous population of cells originating from the bone marrow. As the precursors of dendritic cells, macrophages, and granulocytes, MDSCs can remarkably suppress immune cell responses during tumor immunotherapy. As depicted in Figure S23 (Supporting Information), the percentage of MDSC in tumors treated with mPEI/M1mt decreased to 21.4%, which was 1.7, 1.6, and 1.7‐fold lower than that in tumors treated with PBS, mPEI, and anti‐PD‐L1, respectively. Significantly, the percentage of MDSC in tumors treated with the combination of mPEI/M1mt and anti‐PD‐L1 was decreased to 12.3%, exhibiting strong immune activation. However, tumors treated with the combination of mPEI/M2mt and anti‐PD‐L1 exhibited negligible changes in the percentage of MDSC when compared to tumors treated with PBS. Subsequently, we found that anti‐PD‐L1 combined with mPEI/M1mt increased the percentage of CD4^+^ and CD8^+^ T cells to 38.5% and 30.4%, respectively, which were nearly 3.5 and 5.5 times in comparison with anti‐PD‐L1 alone (10.8% and 5.5%) (Figure 6c). Typically, tumors treated with mPEI/M1mt alone also induced infiltrating a large population of CD4^+^ (20.8%) and CD8^+^ (20.5%) T cells, indicating that mPEI/M1mt mediated polarization of M1 TAMs could induce an antitumor immune response, which was consistent with its high antitumor effect. According to the marking of Foxp3, CD4^+^ can be classified as effective T cells (Teffs, CD4^+^Foxp3^+^) and regulatory T cells (Tregs, CD4^+^Foxp3^−^). Generally, Tregs assist immunosuppressive TME, reversely, Teffs accelerate the antitumor efficiency of CD4^+^ T cells. The population of Tregs was reduced in tumors transplanted with M1mt (32.8%), but not in tumors treated with anti‐PD‐L1 alone (40.3%) (Figure 6d). Additionally, the percentage of Tregs was significantly decreased to 25.6% in mPEI/M1mt treated tumors, which was further low to 18.5% in tumors treated with anti‐PD‐L1 combination with mPEI/M1mt. These results revealed that mPEI/M1mt could improve anti‐PD‐L1 therapy by increasing the tumor infiltration and tumoricidal effects of CD4^+^ and CD8^+^ T cells.

To further elucidate the role of T cell populations and macrophages in enhancing cancer immunotherapy through mPEI/M1mt transplantation, we performed experiments to analyze the effect of mPEI/M1mt + anti‐PD‐L1 on tumor growth in mice after selectively depleted T cells (CD4^+^ or CD8^+^ T cell) or macrophages. The detailed treatment scheme is shown in Figure S24a (Supporting Information). The depletion of T cells (CD8^+^ and CD4^+^ T cells) and macrophages (M2 and M1 TAMs) in the tumor tissues was confirmed by flow cytometric (FCM) analysis (Figure S24b–d, Supporting Information). Although, the depletion of CD8^+^ or CD4^+^ T cells showed negligible effect on M2 and M1 TAMs, the tumor infiltration of CD8^+^ and CD4^+^ T cells decreased to 10.2% and 18.1% upon depletion of macrophages. Meanwhile, the inhibitory effect of mPEI/M1mt + anti‐PD‐L1 on tumor growth was significantly reduced after depletion of T cells (CD4^+^ or CD8^+^ T cell) or macrophages (M2 and M1 TAMs) (Figure S24e–i, Supporting Information). Collectively, these results revealed that the enhanced infiltration of CD4^+^ and CD8^+^ T cells in tumor tissues after mPEI/M1mt transplantation depended on the polarization of M1TAMs. And CD4^+^ and CD8^+^ T cells played a synergistic role in mPEI/M1mt + anti‐PD‐L1 mediated tumor inhibition. Meanwhile, all the mice exhibited negligible changes in body weight demonstrating the negligible systemically toxicity of the treatment strategy (Figure S24j, Supporting Information).

### 2.6 mPEI/M1mt Enhanced Anti‐PD‐L1 Therapy on Murine Colorectal Cancer Models

**Figure 7:**
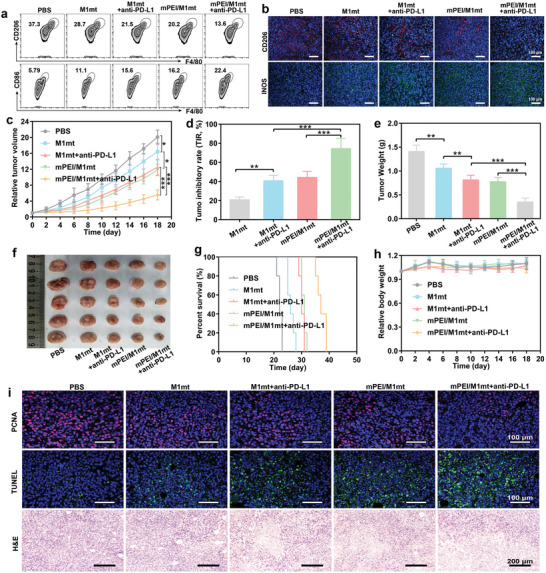
Antitumor effect of CT26 tumor‐bearing mice model in vivo. a) FCM analysis of M1 macrophages (CD11b^+^CD86^+^ F4/80^+^ cells) and M2 macrophages (CD11b^+^CD206^+^ F4/80^+^ cells) in the tumor tissue on day 7 after different treatments. Cells are gated by CD11b^+^ cells, (n = 3). b) Immunofluorescence staining of CD206 and INOS in tumor sections. Mean ± SD (n = 3). c) Tumor growth curves of tumor‐bearing mice under different treatments. d) Tumor inhibitory rate of tumor growth. e) Survival curves of tumor‐bearing mice under different treatments. f) Weight of the tumors harvested on day 18 after different treatments. g) Digital images of tumors collected from mice on day 18 after different treatments. h) The curves of body weight changed over time in tumor‐bearing mice under different treatments. i) Immunofluorescence and H&E staining of tumor sections after different treatments. Mean ± SD (n = 5). *P < 0.05, **P < 0.01, ***P < 0.001.

To investigate whether mPEI/M1mt could potentiate ICB therapy on other tumor types, we established murine colorectal CT26‐bearing mice. After tumor volume reached nearly 50 mm^3^, mice were randomly divided into five groups and received a treatment plan like the 4T1 cell‐bearing mice above. We first revealed that mPEI/M1mt significantly induced the repolarization of M2 TAMs to M1 TAMs. As depicted in **Figure** **7**a, the percentage of M1 TAMs increased to 16.2%, which was 2.8 times in comparison with the PBS group (5.7%). Typically, anti‐PD‐L1 combination with mPEI/M1mt induced the highest population of M1 TAMs (22.4%) and dramatically reduced the number of M2 TAMs (13.6%) in CT26 tumors. The iNOS and CD206 immunofluorescence staining of tumor sections directly further verified that mPEI/M1mt exhibited great performance in mediating M1 TAMs polarization (Figure 7b). To reveal the anticancer efficiency, the tumor size, and mice weight were monitored every two days (Figure S25, Supporting Information). As mentioned above, although mPEI/M1mt alone partially delayed tumor growth, it significantly reduced the tumor burden in combination with anti‐PD‐L1. The RTV in mPEI/M1mt combined with anti‐PD‐L1 reduced to 5.8 which was 0.49 times in comparison with mPEI/M1mt alone (Figure 7c). The highest TIR (Figure 7d), lowest tumor weight (Figure 7e), and smallest tumor tissue (Figure 7f) further verified the excellent anticancer efficiency of mPEI/M1mt combined with anti‐PD‐L1. Undoubtedly, mPEI/M1mt mediated anti‐PD‐L1 dramatically prolonged animal median survival time (Figure 7g), while mice in the PBS group and the other three groups all died within 21–32 days. As depicted in Figure 7h and Figure S26 (Supporting Information), the negligible changes in mice's body weight and histological damage in major organ sections demonstrated the negligible systemic toxicity of all treatments. The proliferation and apoptosis of tumor cells were observed through PCNA and TUNEL staining (Figure 7i). The weakest red fluorescence intensity in PCNA and the strongest green fluorescence intensity in TUNEL directly revealed efficient anticancer therapy of mPEI/M1mt combination with anti‐PD‐L1, which was further verified by most of the tumor cell separation and nuclear ablation. Furthermore, the combination of mPEI/M1mt and anti‐PD‐L1 antibody also can enhance the tumor infiltration of CD8^+^ T cells and tumoricidal effects in CT26 tumor models (Figure S27, Supporting Information). These data demonstrated that targeting transplantation of M1mt is a universal strategy for potentiating anti‐PD‐L1 therapy, which can treat multiple types of tumors including the 4T1 and CT26 tumor models.

## 3 Conclusion

To potentiate ICB cancer therapy, we utilized surface molecularly engineered mitochondria to metabolically repolarize the immunophenotype of M2 TAMs, acquiring an immune‐supportive TME. Mitochondria isolated from M1 macrophages were surface decorated with mannosylated PEI to form mPEI/M1mt for targeted accumulation into M2 macrophages, excellently programming a proinflammatory and tumoricidal M1 phenotype in vitro and in vivo. The mechanism analysis revealed that after transplantation of mPEI/M1mt, the elevated intracellular ROS, induced by resetting the metabolic pattern of M2 macrophages from OXPHOS to glycolysis, stimulated the phosphorylation of NF‐κB p65, MAPK p38 and JNK. After intratumor injection, mPEI/M1mt significantly potentiated anti‐PD‐L1 in combating tumor growth and metastasis by increasing the tumor infiltration and tumoricidal effects of CD4^+^ and CD8^+^ T cells. Notably, as monotherapy, mPEI/M1mt exhibited excellent efficacy in cancer therapy in comparison with anti‐PD‐L1. Together, mPEI/M1mt initiated an adaptive immune response for enhancing ICB therapy by reprogramming pro‐tumoral M2 TAMs to tumoricidal M1 TAMs. Considering that artificial transplantation of active mitochondria has already been used in the preclinic and clinic, the transplantation of mPEI/M1mt not only broadens the scope of mitochondrial transplantation technology but also provides a novel and clinically promising strategy for the treatment of multiple tumor models.

## 4 Experimental Section

### Materials

Mitochondria Isolation Kit, Mito‐Tracker Deep Red probe, Mito‐Tracker Green probe, Griess reagent kit, lipopolysaccharide (LPS), murine IFN‐γ and murine IL‐4 were bought from Beyotime (China). Assay Kits for Agilent Seahorse Analyzers was purchased from Agilent Technologies. Hoechst 33 342 Staining Solution and Annexin V‐FITC/PI Apoptosis Detection Kit were provided by Yeasen (China). Enzyme‐linked immunosorbent assay (ELISA) kit for TNF‐ɑ was purchased from Beijing 4A Biotech Co.Ltd (Beijing, China). RPMI 1640 medium and Fetal bovine serum (FBS) were purchased from Gibco Invitrogen Corp. A specific culture medium for RAW 264.7 was purchased from Wuhan Procell Life Science & Technology Co., LTD. JC‐1 Mitochondrial Membrane Potential Assay Kit was purchased from AAT Bioquest. 75 µm nylon cell strainer and 0.4 µm‐sized Transwell plates were purchased from Corning. Anti‐mouse PD‐L1(B7‐H1) was purchased from Bio X Cell (U.S.A.). All antibodies were purchased from Biolegend.

### Cells and Animals

RAW 264.7 and 4T1 murine breast cancer cells murine macrophages were purchased from Wuhan Procell Life Science & Technology Co., LTD. 4T1 cells were cultured in RPMI 1640 medium containing 1% penicillin‐streptomycin and 10% FBS in an incubator with 5% CO_2_ at 37 °C. RAW 264.7 cells were culture with a specific culture medium.

BALB/c mice (female, 6 weeks) purchased from Beijing HFK Bioscience Co. Ltd, were housed at the SPF care facility with food and water under a 12 h light/12 h dark cycle. All the animal experimental procedures were approved by the Experimental Animal Welfare Ethics Committee, Zhongnan Hospital of Wuhan University (ZN2021232).

### The Synthesis of mPEI

Using polyethyleneimine (PEI_1800_), acrylate (C₄H₆O₂), hydrazine hydrate (N₂H₄·H₂O), and mannose as raw materials, PEI‐NH_2_NH_2_‐Mannose (mPEI) was synthesized according to the following process.

Firstly, compound 1 was prepared using PEI_1800_ and acrylate as follows. Briefly, 1 g PEI_1800_ was dissolved in 10 ml of anhydrous ethanol, and acrylate (1.8 mL) dissolved in 10 mL anhydrous ethanol was added dropwise under a nitrogen atmosphere and stirred for 0.5 h at 0 °C. Then the mixture was continuously stirred for 24 h at 40 °C in a nitrogen atmosphere. After the reaction, the mixture was filtered, and the obtained solution was dropped into anhydrous ether for sedimentation. Finally, the precipitation was collected and dried to obtain the compound 1.

The compound 2 was synthesized from the prepared compound 1 and hydrazine hydrate according to the following steps. In brief, the synthesized compound 1 (1 g) was dissolved in 10 ml anhydrous ethanol, then hydrazine hydrate (5.1 mL) was added, and the mixture was refluxed for 24 h under a nitrogen atmosphere. At the end of reflux, the solution was filtered, collected, put into a dialysis bag (MWCO 500 Da), and dialyzed against ultrapure water for 48 h. The water was exchanged every 6 hours during dialysis. The solution was freeze‐dried to obtain compound 2.

The final product mPEI (compound 3) was obtained by reacting mannose with the synthesized compound 2. Briefly, 1.8 g of mannose was dissolved in 10 mL anhydrous DMSO, and the synthesized compound 2 (1 g) dissolved in 10 mL anhydrous DMSO was added dropwise and stirred for 24 h at room temperature under a nitrogen atmosphere. After the reaction, the mixture was dialyzed against ultrapure water for 48 h using a dialysis bag (MWCO 500 Da). Ultrapure water was exchanged every 6 hours during dialysis. The solution was freeze‐dried to obtain mPEI. All the compounds above were confirmed by ^1^H NMR spectroscopy.

### Preparation of M1 and M2 Macrophage

RAW 264.7 cells were seeded into culture dishes and incubated overnight. Then, the medium was discarded and replaced with the M1 cocktail that contained IFN‐γ (300 ng mL^−1^) and LPS (100 ng mL^−1^) or an M2 cocktail of IL‐4 (10 ng mL^−1^). All cocktails were diluted with fresh medium. The treated cells were incubated for another 24 h to obtain M1 macrophage and M2 macrophage.

### Mitochondrial Labeling

The mitochondria of M1 and M2 macrophages were labeled with a Mito‐Tracker Deep Red probe. Briefly, Cells were incubated with a Mito‐Tracker Deep Red probe working solution (100 nM) for 20 min at 37 °C in an incubator. After incubation, the medium was removed, and cells were washed three times with PBS. The labeling condition of mitochondria was observed by a Laser confocal scanning microscope (CLSM).

### Mitochondrial Isolation

The mitochondria of M1 macrophage and M2 macrophage were isolated using a commercial Mitochondria Isolation Kit. The isolation procedures were performed according to the protocol. Mitochondria isolated from macrophages were termed Mito, Mitochondria isolated from M1 and M2 macrophages were named M1mt and M2mt, separately, and mitochondria isolated from various macrophages all can be called Mito. All the Mito were maintained at 4 °C or stored at −80 °C before downstream processing. Mitochondrial surface modification

To reverse the surface charge of the isolated mitochondria, positively charged mPEI was used to modify the isolated mitochondria. Briefly, M1mt and M2mt dispersed in PBS were added with mPEI (20, 50, 80 µg mL^−1^) and incubated for 2 min at room temperature. After incubation, the precipitation was collected by centrifugation (8000 g, 10 min) and washed three times with PBS to obtain the mPEI‐modified M1mt (mPEI/M1mt) and M2mt (mPEI/M2mt).

### Characterization of the Isolated Mitochondria and Modified Mitochondria

The size distribution and zeta potential of M1Mt, M2Mt, mPEI/M1mt, and mPEI/M2mt were examined by ZETA‐SIZER Nano Series ZEN3600 (Malvern Instruments Ltd., UK). The morphology was observed by transmission electron microscopy (TEM, JEM‐100CXII 100 kV, Tokyo, Japan).

### Detection of Mitochondrion Membrane Potential

A JC‐1 dye was used to evaluate the integrity of isolated mitochondria by detecting the mitochondrial membrane potential. Briefly, M1mt, M2mt, mPEI/M1mt, and mPEI/M2mt were incubated with 200 µL of JC‐1 working solution (20 µM) for 30 min at 37 °C. M1mt and M2mt treated with 5 mM 1 µL CCCP were served as positive control. After staining, mitochondria were centrifuged (8000 g, 5 min) and resuspended in 200 µL PBS for further analysis by flow cytometry (FCM). The ratio of red/green fluorescence intensity was calculated to evaluate the integrity of mitochondria.

### Cellular uptake in vitro

The cellular uptake of isolated mitochondria was evaluated by confocal laser scanning microscope (CLSM) and FCM. M2 macrophages and 4T1 cells were seeded into confocal dishes or 6‐well plates with a density of 1.0×10^5^ cells/well. After overnight incubation, M2mt, mPEI/M2mt, M1mt, and mPEI/M1mt (Mito, 5 ×10^6^ /mL) were added and incubated for 1 h or 4 h. After incubation, the medium was removed and cells were washed with PBS. For CLSM, cells were stained with Hoechst 33 342 (5 nM) for 10 min and observed by CLSM. As for FCM analysis, cells were collected and analyzed by FCM.

To evaluate the effect of mannose on the cell uptake of mPEI‐modified Mito, M2 macrophages were pretreated with sufficient mannose for 1 h before the addition of Mito.

### In Vitro Cytokines Measurements

The content of TNF‐ɑ, NO, IFN‐γ, IL‐1, IL‐10, and TGF‐β1 generated by mitochondria‐treated M2 macrophages was determined by the corresponding ELISA Kit. Briefly, M2 macrophages seeded on 6‐well plates and adhered overnight were added with different concentrations of mPEI, M1mt, M2mt, mPEI/M1mt, and mPEI/M2mt (Mito, 2.5 ×10^6^, 5×10^6^, 10×10^6^ /mL). At the predetermined time point (8 h, 12 h, and 24 h), supernatants were collected, and the content of TNF‐ɑ and NO was detected according to the recommended procedure. The content of IFN‐γ, IL‐1, IL‐10, and TGF‐β1 in the supernatant was detected after different treatments (Mito, 5 ×10^6^) for 24 h.

### Intracellular ROS measurement

The generation of ROS was evaluated by CLSM and FCM. In brief, M2 macrophages were seeded into confocal dishes or 6‐well plates and cultured for 24 h. The following day, cells were added with different mitochondrial (mPEI, M1mt, M2mt, mPEI/M1mt, and mPEI/M2mt (Mito, 5 ×10^6^ /mL). After 24 h co‐culture, cells were washed with serum‐free medium for 3 times and stained with DCFH‐DA (10 µM) for 30 min at 37 °C. After removing the dyeing solution and washing with serum‐free medium, the nucleus of cells was stained with Hoechst 33 342 (5 µg mL^−1^) and then observed by CLSM. As for FCM analysis, cells were collected, stained with DCFH‐DA, and analyzed by FCM.

### In vitro CD80, CD86, CD206 Detection

To evaluate the repolarization efficiency of the isolated mitochondria on M2 macrophages repolarized to M1 macrophages, the M1 markers (CD80, CD86) and M2 marker (CD206) were analyzed by FCM and CLSM. As for FCM, M2 macrophages were seeded into 6‐well plates and adhered overnight, followed by the addition of mPEI, M1mt, M2mt, mPEI/M1mt, and mPEI/M2mt (Mito, 5 ×10^6^/mL) and incubation for 24 h. After incubation, cells were washed, collected, and stained with fluorescent‐conjugated CD80, CD86, and CD206 antibodies at 4 °C for 25 minutes. After staining, cells were analyzed by FCM. As for CLSM, cells were seeded into confocal dishes, treated with various mitochondria, stained with CD86 antibodies and Hoechst 33 342, and observed by CLSM.

### Mitochondrial Membrane Potential of M2 Macrophages

JC‐1 dye was used to detect the mitochondrial membrane potential of M2 macrophages after different treatments. Briefly, M2 macrophages were seeded into confocal dishes and incubated overnight. The following day, cells were treated with different materials of mPEI, M2mt, mPEI/M2mt, M1mt, and mPEI/M1mt (Mito, 5 ×10^6^/mL) for 24 h, respectively. After incubation, cells were stained with JC‐1 dying solution (20 µM) for 20 minutes, washed with PBS, and observed by CLSM. The ratio of Red/Green fluorescence intensity was analyzed by Image J.

### Preparation of Mito Treated‐M2 Macrophages

M2 macrophages treated with mPEI/M1mt for 24 h were termed mPEI/M1mt‐M2ø, M2ø treated with other mitochondria was named similarly. Briefly, M2 macrophages were seeded into the culture plates and cultured overnight. Then, cells were added with mPEI, M2mt, mPEI/M2mt, M1mt, mPEI/M1mt (Mito: 5 × 10^6^/mL) and incubated for 24 h. After incubation, mPEI/‐M2ø, M2mt‐M2ø, mPEI/M2mt‐M2ø, M1mt‐M2ø and mPEI/M1mt‐M2ø were obtained, respectively. And Mito treated M2ø was named Mito‐M2ø.

### Intracellular Distribution Study

M2 macrophages were cultured in confocal laser dishes (1×10^5^cells per dish) overnight. Mito‐Tracker Red labeling M1mt and mPEI/M1mt (Mito, 5 ×10^6^ /mL) were separately added into the medium and then incubated for 1 h, 4 h, and 8 h. Subsequently, the cells were washed with PBS three times and then stained with 250 nM Lysotracker Green for 30 min at 37 °C in the dark. After washing with PBS three times, the cells were immediately observed using CLSM. The green fluorescence of Lysotracker Green and red fluorescence of Mitos were separately detected using 488/525 nm and 561/600 nm excitation/emission filters.

### In Vitro Cytotoxicity Assay

The cytotoxicity of Mito on M2 macrophages was evaluated by MTT assay. Briefly, M2 macrophages were seeded into 96‐well plates with a density of 3×10^3^ cells/well and incubated overnight. Then, cells were added with different materials (mPEI, M1mt, M2mt, mPEI/M1mt, and mPEI/M2mt) with various concentrations (Mito,0.1 × 10^6^ – 10 × 10^6^/mL) and incubated for another 24 h. After incubation, the medium was removed, cells were washed with PBS, and the MTT in fresh medium was added. After incubating with MTT for another 4 h, all the medium was discarded, and 150 µL DMSO was added to dissolve the formazan. The absorbance of each well at 570 nm was measured by a microplate reader (Thermo Scientific, USA). The cell viability of M2 macrophages was calculated as follows (n = 3):

(1) $$\mathrm{Cell}\;\mathrm{viability}\left(\%\right)=\left[\left(A_{\mathrm{sample}}-A_{0}\right)/\left(A_{\mathrm{control}}-A_{0}\right)\right]\times 100\%$$

where A represents the absorbance at 570 nm.

The cytotoxicity of Mito‐M2Mø on 4T1 cells was evaluated by MTT assay. Briefly, 4T1 cells were seeded into 96‐well plates with a density of 3 × 10^3^ cells per well. The following day, different kinds of mitochondria‐treated M2Mø (M2Mø, mPEI‐M2Mø, M2mt‐M2Mø, mPEI/M2mt‐M2Mø, M1mt‐M2Mø, and mPEI/M1mt‐M2Mø) with a density of 1×10^4^ cells/well were added and incubated for 24 h. Meanwhile, different mitochondria‐treated M2Møs were seeded into new 96‐well plates (10^4^ cells/well) to set as a background. Then, cells were treated with MTT referr*ing to the above metho*d. The cell viability of 4T1 cells was counted as follows (n = 3):

(2) $$\begin{array}{ccc} \mathrm{Cell}\;\mathrm{viability}\left(\%\right) & = & [\left(A_{\left(4T1\;\mathrm{cells}+\mathrm{different}\;M2Møs\right)}-A_{\left(\mathrm{different}\;M2Møs\right)}-A_{0}\right)/\\ & & \left(A_{\mathrm{control}}-A_{0}\right)\;]\times 100\% \end{array}$$

where A represents the absorbance at 570 nm, A_(4T1 cells+different M2Møs)_ represents the sample of 4T1 cells treated with different kinds M2Mø, represents the absorbance of the sample of 4T1 cells treated with different treated M2Mø, A_(different M2Møs)_ represents the absorbance the sample of different kinds of M2Mø seeded in the new plates, A_control_ represents the absorbance of the sample of 4T1 cells treated with PBS.

### Apoptosis Assay

The apoptosis of 4T1 cells induced by Mito‐M2Mø was evaluated by an Annexin V‐FITC/PI Apoptosis Detection Kit. Briefly, 4T1 cells were seeded into the bottom wells of a 0.4 µm‐sized 6‐well transwell system with a density of 1 × 10^5^ cells/well. After 24 h, 3×10^5^ cells/well of mPEI‐M2ø, M2mt‐M2ø, mPEI/M2mt‐M2ø, M1mt‐M2ø, and mPEI/M1mt‐M2ø were added into the upper wells and co‐incubation for 24 h. 4T1 cells in the bottom wells were collected, washed, centrifuged, and stained with 200 µL working solution containing Annexin V‐FITC/PI for 10 min at room temperature. After staining, cells were analyzed by FCM immediately.

### Western Blotting Analysis

Western blotting (WB) was performed to evaluate the expression of p‐STAT1, p‐p38, and p‐p65 after different treatments. Briefly, cells were seeded into 6‐well plates and incubated overnight. Then, mPEI/M1mt, and mPEI/M1mt + ROS scavenger was added and incubated for 24 h. After incubation, cells were lysed, and proteins were obtained for western blotting analysis.

### Seahorse Analysis

The extracellular acidification rates (ECAR) and oxygen consumption rates (OCR) in M2 macrophages were examined using a Seahorse XF24 extracellular flux analyzer (Agilent) to determine the repolarization effect on M2 macrophages of mPEI/M1mt. The experiment was conducted according to the manufacturer's protocol. Briefly, 2 days before the experiment, M2 macrophages were seeded into an XF24 cell plate and incubated in a 5% CO_2_ incubator at 37 °C overnight. The following day, cells received different treatments (PBS, M2mt, mPEI/M2mt, M1mt, mPEI/M1mt, mPEI/M1mt + ROS scavenger, and IFN‐γ + LPS; Mito: 5×10^6^/mL, IFN‐γ: 300 ng mL^−1^, LPS: 100 ng mL^−1^) and co‐incubated for 24 h. Meanwhile, 200 µL of Seahorse XF calibration solution was added to the utility plate and the hydration probe was placed in a 37 ° C incubator without CO_2_ overnight to hydrate the probe. On the day of the experiment, ECAR and OCR analysis were conducted. In OCR analysis, the cell culture medium was replaced with an XF assay medium (containing 1 mM sodium pyruvate, 10 mM glucose, and 2 mM glutamine, pH adjusted to 7.4) and incubated in an incubator without CO_2_ at 37 ° C for 1 h. During the analysis process, oligomycin (1 µM), carbonyl cyanide‐4 (trifluoromethoxy) phenylhydrazone (FCCP, 0.5 µM), and rotenone/antimycin A (1 µM) were sequentially injected according to the fixed procedure. As for ECAR, the cell culture medium was replaced with an XF assay medium (containing 2 mmol/L l‐glutamine, pH adjusted to 7.4) and placed in a non‐CO_2_ incubator at 37 ° C for 1 h. During the analysis process, glucose (10 mM), oligomycin (1 µM), and 2‐deoxyglucose (2‐GD, 50 mM) were sequentially added according to the fixed procedure. The data were collected using the XFe24 wave software.

### Antitumor Effect In Vivo

BALB/c mice were applied to determine the antitumor effect in vivo. To establish the 4T1 cells‐bearing mice model, 4T1 cells (2×10^6^) suspended in 100 µL PBS were injected subcutaneously into the right side of a mouse's back. When tumor volume reached approximately 50 mm^3^, mice were randomly divided into 8 groups as follows (n = 6): 1) PBS; 2) mPEI; 3) anti‐PD‐L1; 4) mPEI/M2mt + anti‐PD‐L1; 5) M1mt; 6) M1mt + anti‐PD‐L1; 7) mPEI/M1mt; and 8) mPEI/M1mt + anti‐PD‐L1, n = 6, and then administrated with different treatment strategies. Anti‐PD‐L1 antibodies were injected 1 day after the Mito injection. Specifically, mPEI and pure or modified Mito were locally injected into the tumor on day 0, day 2, and day 4 (Mitos 5×10^6^), and anti‐PD‐L1 antibody (100 µL in PBS, 3 mg k^−1^g each time) was intravenously injected on day 1, day 3, and day 5. The tumor volume and body weight of each mouse were monitored and recorded every two days for 18 days. At the end of the experiment, mice were sacrificed, and tumors were collected, weighed, and pictured.

Tumor volume was calculated as follows: V = L×W^2^/2, Where V represents tumor volume, and “L” and “W” represent the length and width of the tumor, separately. Tumor inhibition rate (TIR) was calculated as follows: TIR = [1‐(V_tf_‐V_ti_)/(V_pf_‐V_pi_)] *100%, where V_pf_ and V_pi_ represent the final and initial tumor volumes of the mice with PBS treatment, and V_tf,_ and V_ti_ represent the final and initial tumor volumes of the mice with other treatments.

As for the CT26 cell‐bearing mice model, mice were subcutaneously injected with CT26 cells (2×10^6^ cells in 100 µL PBS) on the right side of the back. When tumor volume reached approximately 50 mm^3^, mice were randomly divided into 5 groups as follows (n = 6): 1) PBS; 2) M1mt; 3) M1mt + anti‐PD‐L1; 4) mPEI/M1mt; and 5) mPEI/M1mt + anti‐PD‐L1, n = 6, and then administrated with different treatment strategies. In subsequent treatment, CT26 cell‐bearing mice were monitored and treated like the 4T1 cell‐bearing mice above.

### Immune Cells Analysis in the Tumor Microenvironment

On day 7 of the treatment, mice of each group were sacrificed, and tumor tissues and tumor‐draining lymph nodes (TDLNs) were collected. Tumor tissues were digested into single cells using a digestive formula of 2% FBS 1640 medium containing hyaluronidase (1 mg mL^−1^), collagenase IV (0.1 mg mL^−1^), and DNase I. After being blocked with Fc receptor blocker anti‐CD16/32 antibodies for 15 min at 4 °C, cells were stained with different antibodies and analyzed by FCM to evaluate the proportion of M1 macrophages, M2 macrophages, CD8^+^ T cells, and Tregs in the tumor. To detect the proportion of M1 macrophages, cells were stained with FITC anti‐mouse CD11b, PE anti‐mouse F4/80, and APC anti‐mouse CD86 for 20 min at 4 °C. As for M2 macrophages, the cell surface markers were firstly stained with FITC anti‐mouse CD11b and PE anti‐mouse F4/80 for 20 min at 4 °C, followed by the intracellular staining of APC anti‐mouse CD206 antibody for 20 min at room temperature after fixation and permeabilization with 4% paraformaldehyde and perm wash buffer. To analyze the Treg, cells were stained with APC‐anti‐mouse CD25 and FITC‐anti‐mouse CD4, fixed, permeabilized, and stained with PE‐anti‐mouse FOXP3.

As for evaluating the antitumor efficacy of mPEI/M1mt+aanti‐PD‐L1 mice after immune cell depletion, mice were randomly divided into groups G1 to G5 when the tumor volume reached approximately 80 mm^3^, including G1: PBS, G2: mPEI/M1mt+anti‐PD‐L1+anti‐CD8 antibody, G3: mPEI/M1mt+anti‐PD‐L1+anti‐CD4 antibody, G4: mPEI/M1mt+anti‐PD‐L1+macrophages scavenger, G5: mPEI/M1mt+anti‐PD‐L1, and then subjected to different treatments as required. Briefly, anti‐CD4 (5 mg k^−1^g), anti‐CD8 (5 mg k^−1^g), and macrophage scavenger (Clophosome‐A, 2 mg k^−1^g) were administered via tail vein on day 0, followed by mPEI/M1Mt was locally injected into tumor tissues on day 1, and PD‐L1 administration via tail vein injection on day 2. Tumor size and mouse body weight were monitored according to the aforementioned methods.

### Evaluation of the Activated CD8+T Cells and DC Maturation in TDLNs

TDLNs were squeezed with a syringe and dispersed into single cells. Cells stained with APC anti‐mouse CD11c, FITC anti‐mouse CD80, and PE anti‐mouse CD86 for 20 min at 4 °C to evaluate dendritic cell (DC) maturation. For the analysis of activated CD8^+^ T cells, cells were incubated with FITC anti‐mouse CD3, APC anti‐mouse CD8, and PE anti‐mouse CD69 for 20 min at 4 °C. For MDSCs analysis, cells were stained with APC‐anti‐CD45, FITC‐anti‐CD11b, and PE‐anti‐GR‐1 antibodies.

### H&E and Immunofluorescence Staining

On day 7 of the treatment, mice were sacrificed, and tumor tissues and major organs (heart, liver, spleen, lung, and kidney) were collected, fixed with 4% paraformaldehyde, embedded in paraffin, sliced, and stained. Slices stained with H&E were aimed to observe the histopathological damage of tumors and organs after treatment.

The apoptosis and proliferation in tumor tissues after treatments were detected by terminal‐deoxynucleotidyl transferase‐mediated nick end labeling (TUNEL) and proliferating cell nuclear antigen (PCNA) methods. Meanwhile, the expression of INOS, CD206, CD80, and CD86 in tumor sections was detected to evaluate the phenotype of macrophages in tumors after different treatments. The expression of TNF‐ɑ in tumor tissues was also detected. The expression of CD31 was applied to observe the vessels of tumor tissues after treatment.

### Statistical Analysis

All the statistical analysis was performed using GraphPad Prism 7.0. The statistical data were presented as Mean + SD. The student's t‐test was used to analyze the differences between the two groups. A statistical significance of P < 0.05 was selected, and * indicated P < 0.05, ** indicated P < 0.01, and *** indicated P < 0.001, respectively.

## Conflict of Interest

The authors declare no conflict of interest.

## Supporting information

Supporting Information

## Data Availability

Research data are not shared.
