# Supplementary material for: Surface Molecularly Engineered Mitochondria Conduct Immunophenotype Repolarization of Tumor‐Associated Macrophages to Potentiate Cancer Immunotherapy
Source: Adv Sci (Weinh). 2024 Aug 9;11(38):2403044. doi: 10.1002/advs.202403044 (PMC11481252; doi:10.1002/advs.202403044)
Supplement: Supplementary file 1 — Supporting Information [file ADVS-11-2403044-s001.pdf]

ADVANCED SCIENCE
Open Access

Supporting Information

for *Adv. Sci.*, DOI 10.1002/advs.202403044

Surface Molecularly Engineered Mitochondria Conduct Immunophenotype Repolarization of Tumor-Associated Macrophages to Potentiate Cancer Immunotherapy

Cai-Ju Zhang, Jia-Mi Li, Dan Xu, Dan-Dan Wang, Ming-Hui Qi, Feng Chen, Bo Wu, Kai Deng and Shi-Wen Huang**

Surface molecularly engineered mitochondria conduct immunophenotype repolarization of tumor-associated macrophages to potentiate cancer immunotherapy

Cai-Ju Zhang, Jia-Mi Li, Dan Xu, Dan-Dan Wang, Ming-Hui Qi, Feng Chen, Bo Wu, Kai Deng, Shi-Wen Huang**

C. Zhang, B. Wu, K. Deng
Department of Radiology, Zhongnan Hospital of Wuhan University, Wuhan University, Wuhan 430071, China
E-mail: deng-k@whu.edu.cn

J. Li, D. Wang, M. Qi
Key Laboratory of Biomedical Polymers of Ministry of Education, Department of Chemistry, Wuhan University, Wuhan 430072, China.

J. Li
Department of Radiology, Renmin Hospital of Wuhan University, Jiefang Road 238,Wuchang District, Wuhan, Hubei 430060, China

D. Xu
Department of Nuclear Medicine, Zhongnan Hospital of Wuhan University, Wuhan University, Wuhan 430071, China

C. Zhang, F. Chen
Department of Radiology, Hainan Hospital Affiliated to Hainan Medical University, Hainan 570311, China

S. Huang

Department of Orthopedic Trauma and Microsurgery, Zhongnan Hospital of Wuhan University, Wuhan 430071, China
Key Laboratory of Biomedical Polymers of Ministry of Education, Department of Chemistry, Wuhan University, Wuhan 430072, China.
E-mail: swhuang@whu.edu.cn

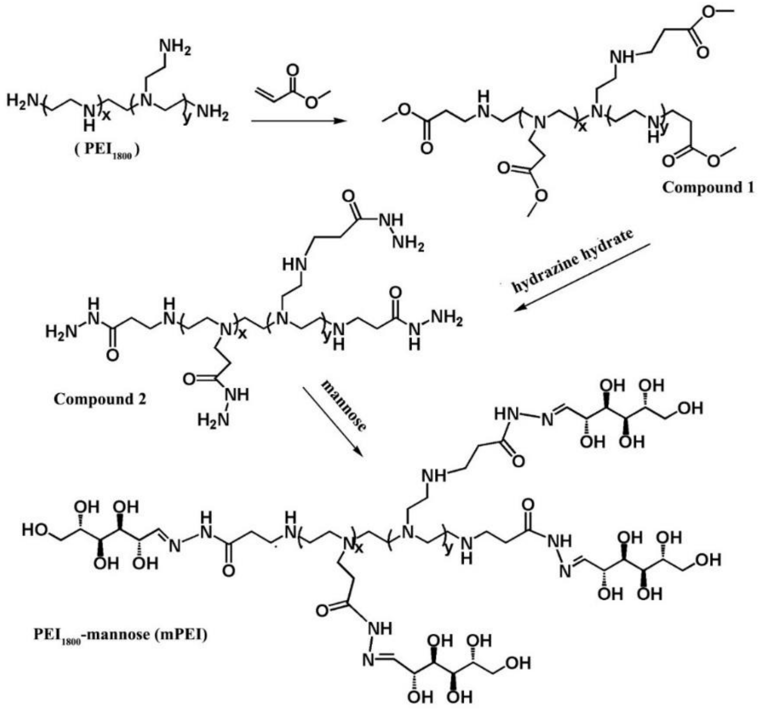

Figure S1. Synthesis procedures of mannosylated PEI (mPEI)

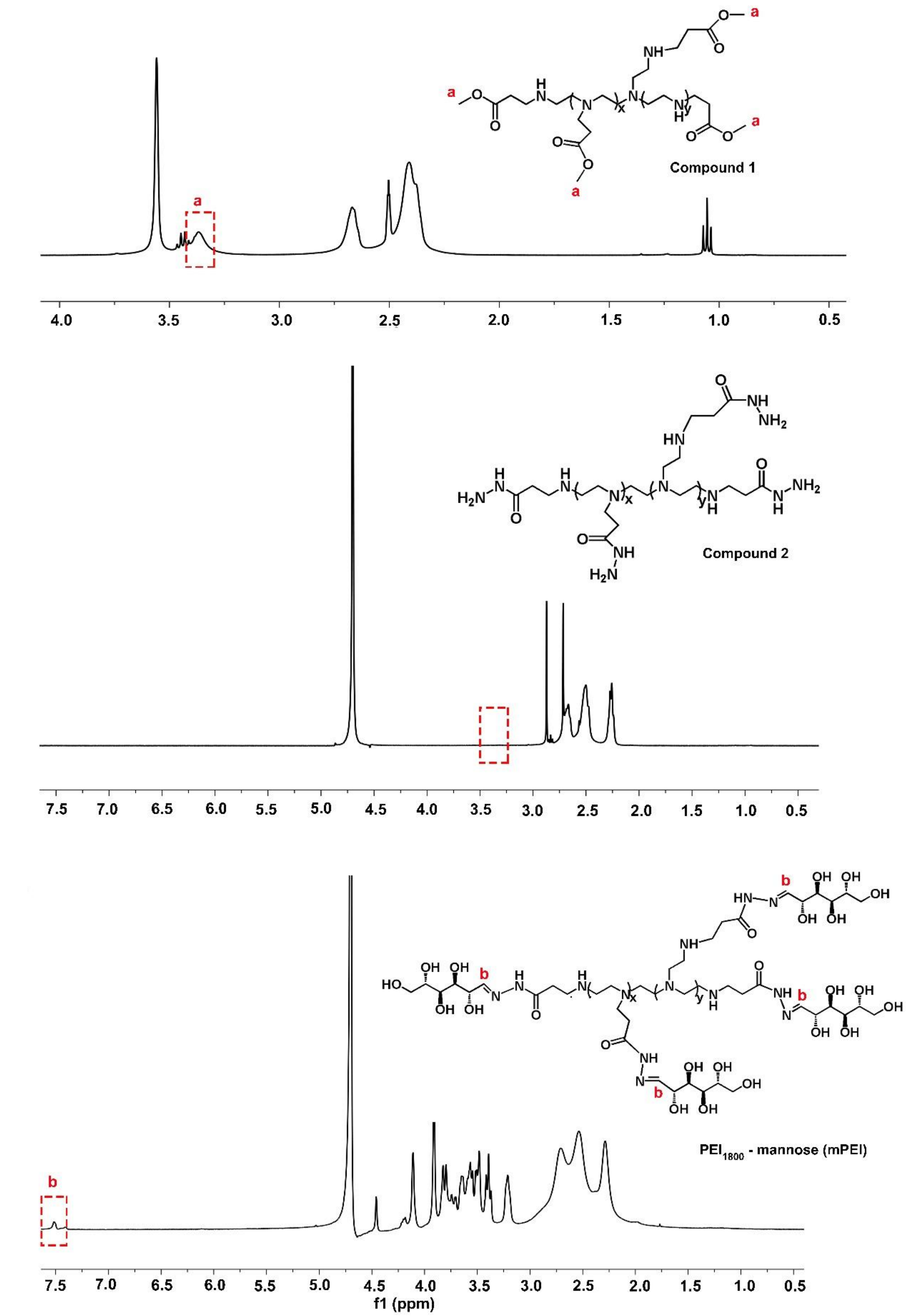

Figure S2. ^{1}H NMR spectrum of Compound 1 in DMSO-d_6, Compound 2 and mPEI in D_2O

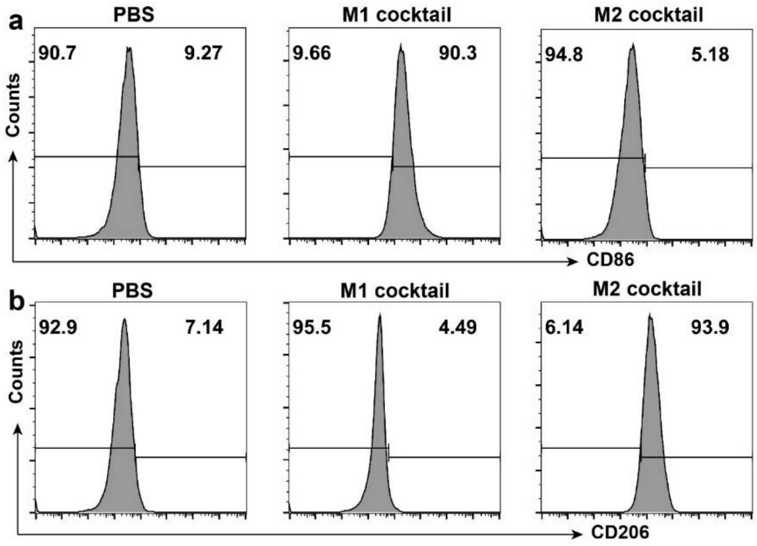

Figure S3. FCM analysis of the expression of (a) CD86 and (b) CD206 of RAW 264.7 after being treated with an inflammatory cocktail interferon-γ (IFN-γ) and lipopolysaccharides (LPS), and anti-inflammatory cytokine IL-4.

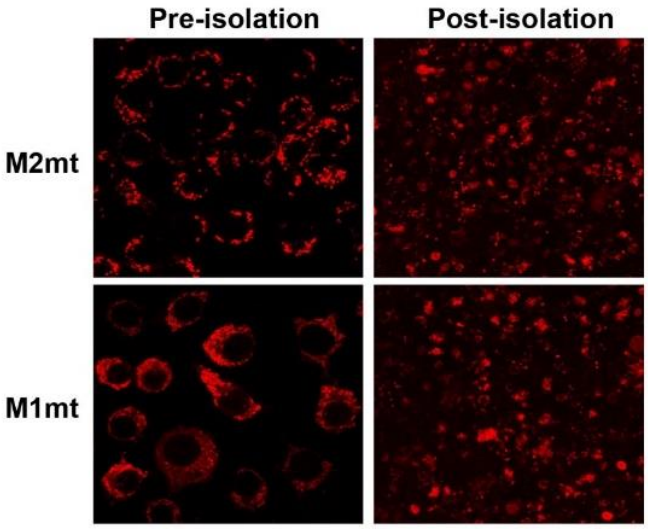

Figure S4. CLSM images of Mito Tracer Deep Red probe labeled mitochondria of M1 cells and M2 cells before and after isolation.

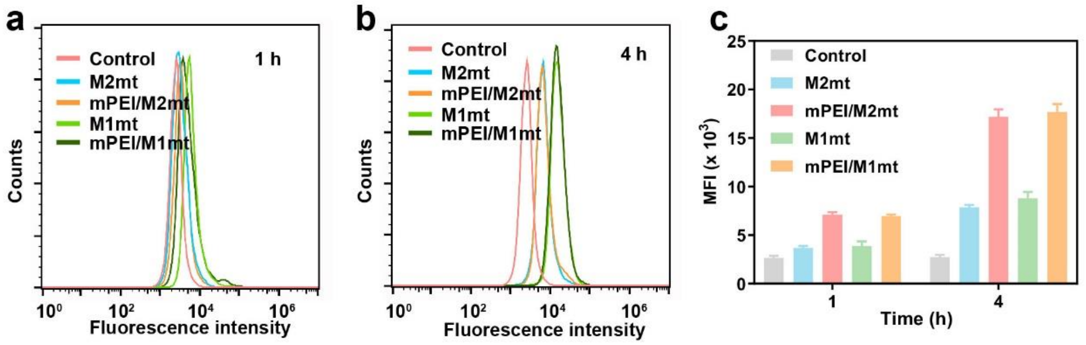

Figure S5. (a-b) Flow cytometry (FCM) analysis and **(c)** the corresponding quantification of cellular uptake of M2mt, mPEI/M2mt, M1mt, and mPEI/M1mt (labeled with Mito Tracker Deep Red) by M2 macrophages after incubated for 1 h and 4 h.

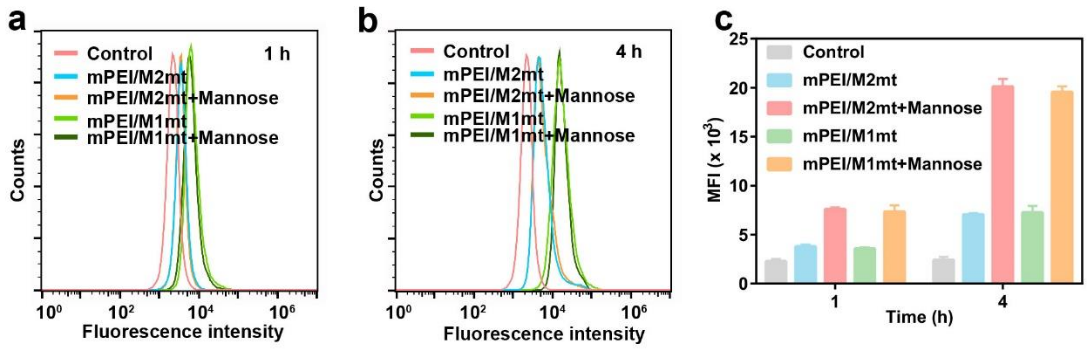

Figure S6. **(a-b)** Flow cytometry analysis and **(c)** quantification of cellular uptake of mPEI/M2mt and mPEI/M1mt by M2 macrophages pretreated with or without sufficient mannose. M2 macrophages were pretreated with or without mannose and then incubated with mPEI/M2mt, and mPEI/M1mt (Mito Tracker Deep Red labeling) for 1 h and 4 h. Mean ± SD (n=3).

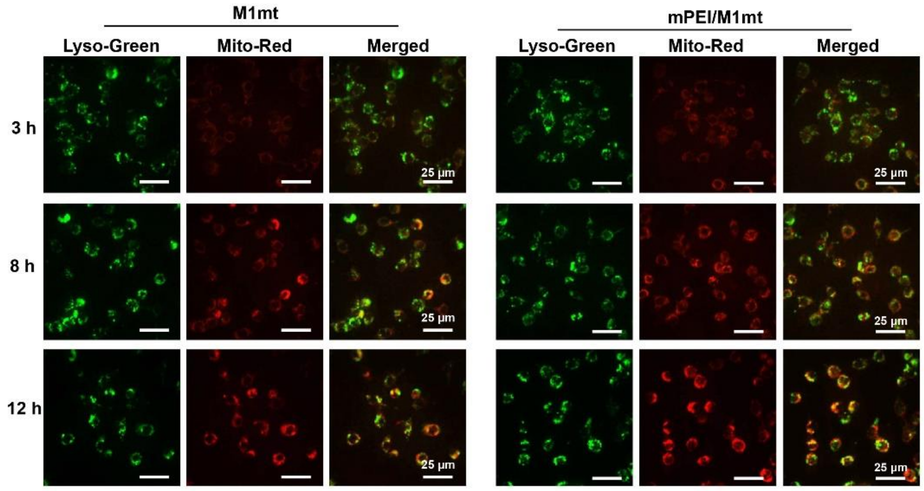

Figure S7. The co-localization of M1mt and mPEI/M1mt with lysosome in M2 macrophages was observed by CLSM. M2 cells were incubated with M1mt and mPEI/M1mt (Mito Tracker Deep Red labeling) for 3 h, 8 h, and 12 h, then stained with lyso-Tracker Green to label lysosomes before observation.

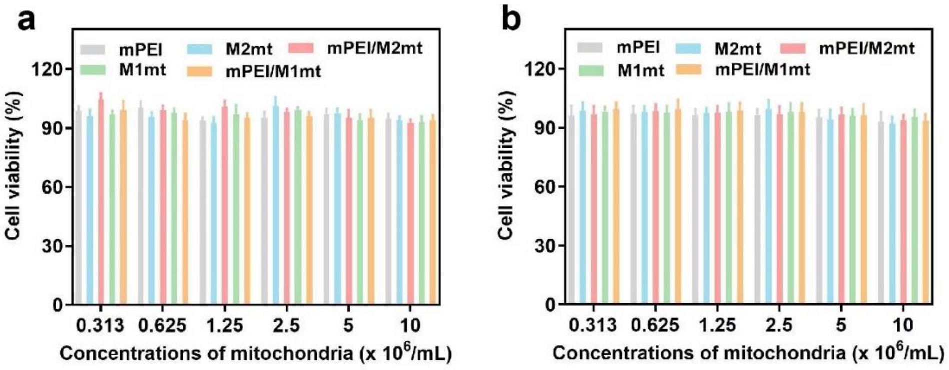

Figure S8. Cell viability of **(a)** M2 cells and **(b)** 4T1 cells after being treated with different concentrations of PBS, mPEI, M2mt, mPEI/M2mt, M2mt, and mPEI/M1mt for 24 h. Mean ± SD (n=3).

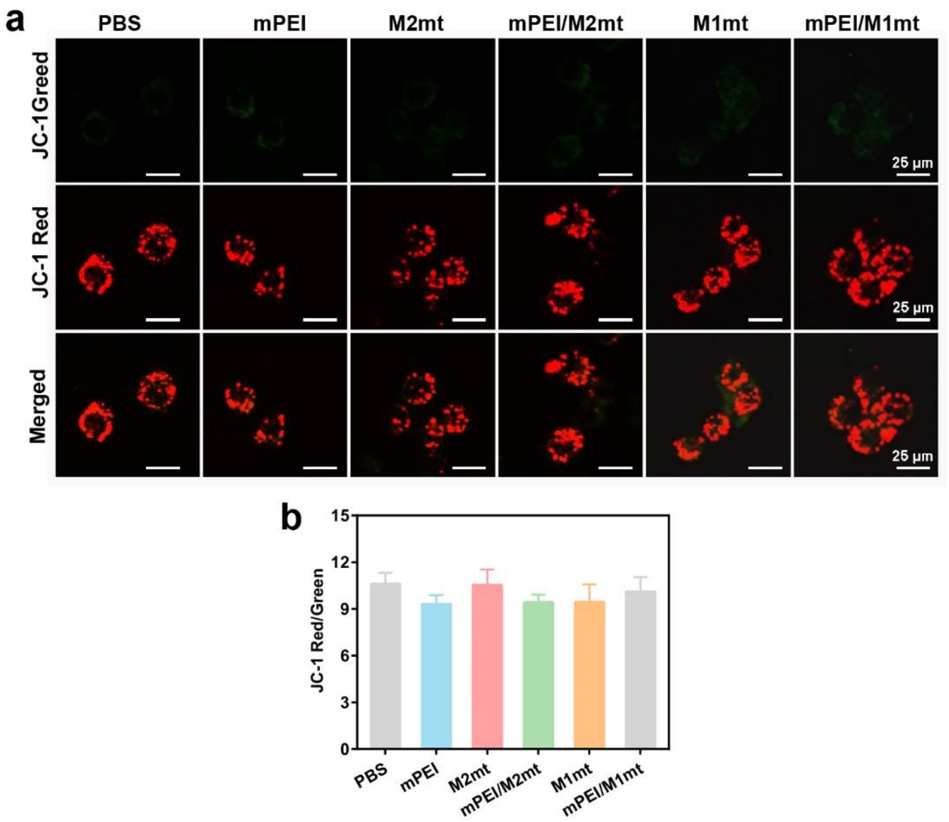

Figure S9. Detection of mitochondrial viability. (a) CLSM images and (b) the corresponding JC-1 Red/Green ratio of M2 cells after treatment with PBS, mPEI, M2mt, mPEI/M2mt, M2mt, and mPEI/M1mt for 24 h. Mean ± SD (n=3).

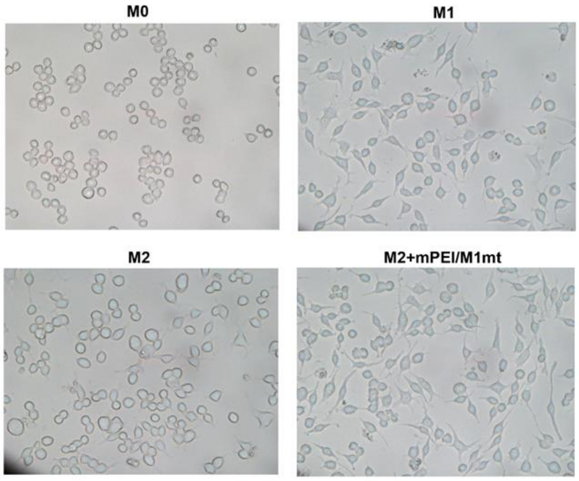

Figure S10. Morphology of macrophages after different treatments. M1: M0 treated with IFN-γ and LPS for 24 h; M2: M0 treated with IL-4 for 24 h; M2+mPEI/M1mt: M2 treated with mPEI/M1mt for 24 h.

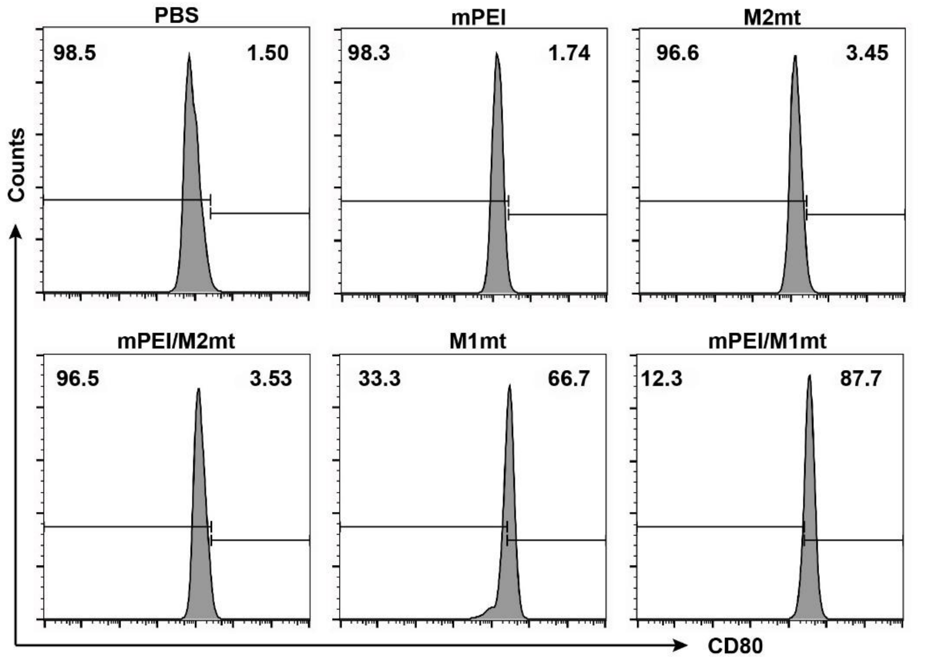

Figure S11. Flow cytometry analysis of the expression of CD80 in M2 cells after different treatments for 24 h. n=3.

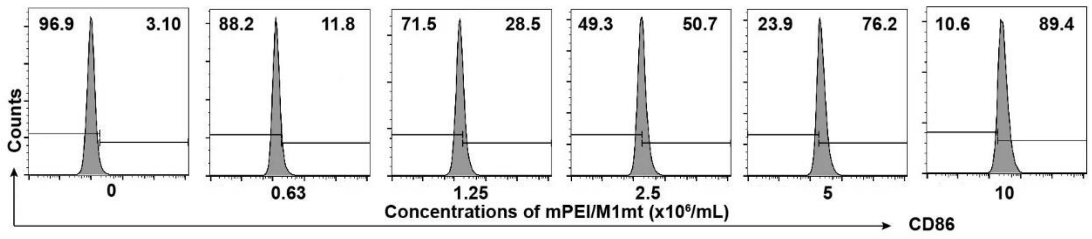

Figure S12. FCM analysis of CD86 in M2 macrophages after being treated with various concentrations of mPEI/M1mt. n=3.

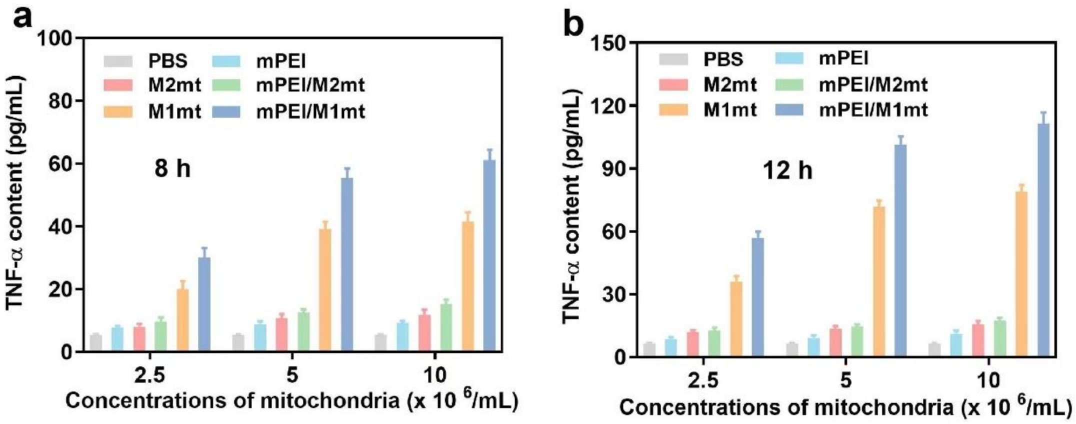

Figure S13. Production of TNF-α in the supernatant of M2 cells that incubated with different concentrations of mPEI, M2mt, mPEI/M2mt, M1mt, and mPEI/M1mt for (a) 8 h and (b) 12 h. Mean ± SD (n=3).

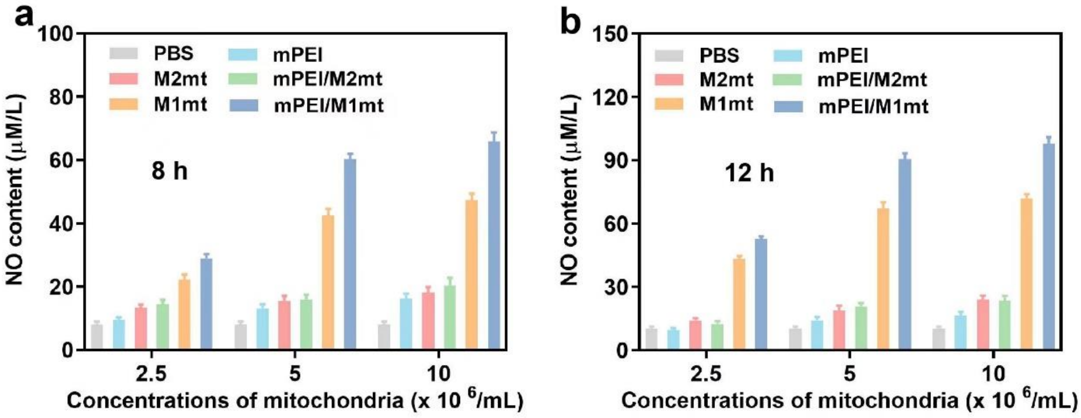

Figure S14. Production of NO in the supernatant of M2 cells that incubated with different concentrations of mPEI, M2mt, mPEI/M2mt, M1mt, and mPEI/M1mt for (a) 8 h and (b) 12 h. Mean ± SD (n=3).

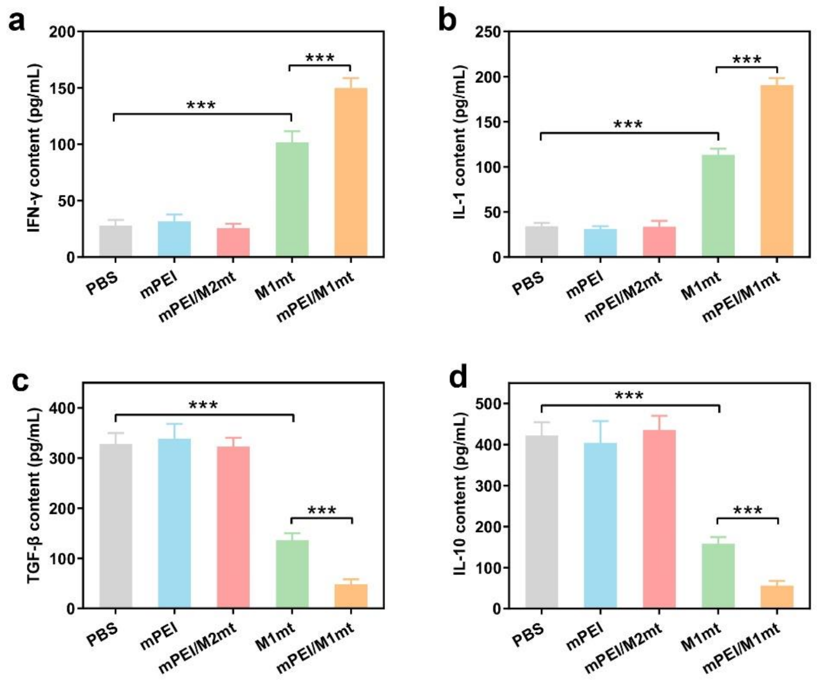

Figure S15. Production of the IFN-γ, IL-1, IL-10, and TGF-β in the supernatant of M2 macrophages after incubating with mPEI, M2mt, mPEI/M2mt, M1mt, and mPEI/M1mt for 24h at a Mitochondria concentration with 5 × 10^6/mL. Mean±SD, n=3, *P < 0.05; **P<0.01; ***P<0.001.

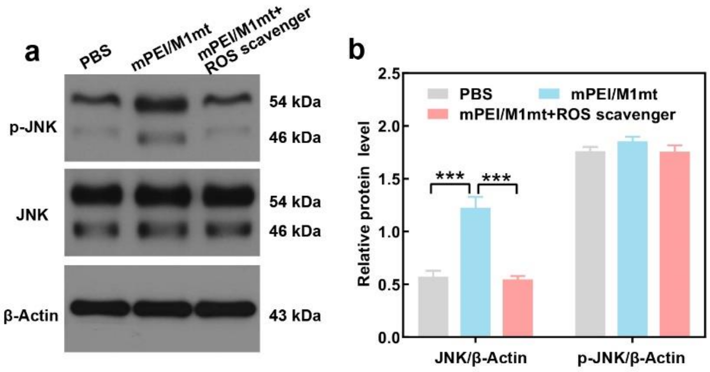

Figure S16. (a) Western blotting analysis and (B) corresponding relative protein level of p-JNK expression in M2 macrophages treated with mPEI/M1mt in the presence and absence of ROS-scavenger N-acetylcysteine. Mean±SD, n=3, *P < 0.05; **P<0.01; ***P<0.001.

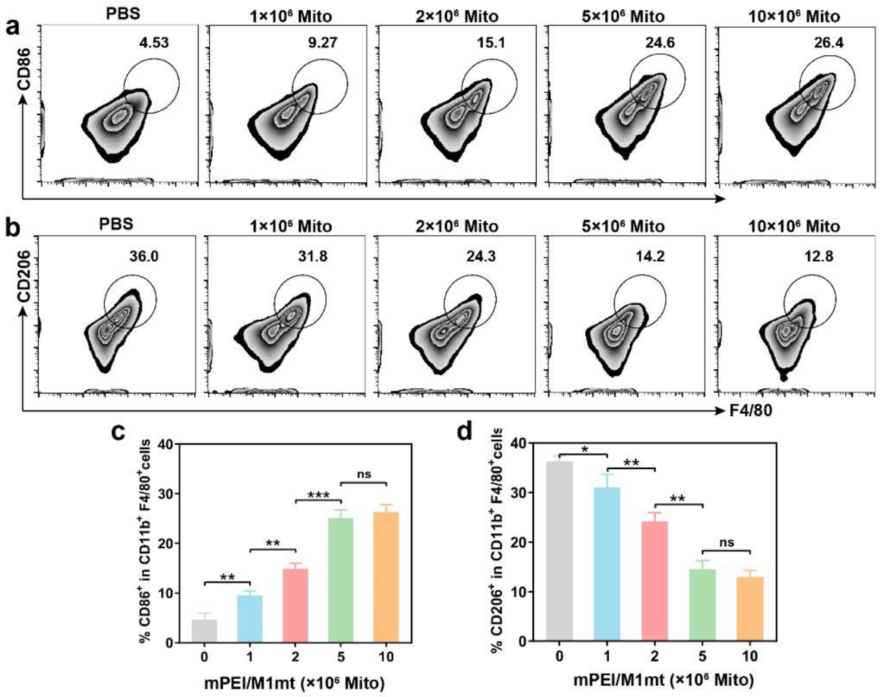

Figure S17. FCM analysis and corresponding statistical data of (a, c) M1 macrophages (CD86+ F4/80+ cells, gated by CD11b+ cells) and (b, d) M2 macrophages (CD206+ F4/80+ cells, gated by CD11b+ cells) in the tumor tissue on day 7 after different treatments. The mPEI/M1mt with various doses (0, 1, 2, 5, and 10 $\times 10^6$ Mitos each time) was locally injected into tumor tissues on day 0, day 2, and day 4, and anti-PD-L1 antibody was intravenously injected on day 1, day 3, day 5 (100 μL in PBS, 3 mg/kg each time). Mean±SD (n=3)，ns: no significance, *P<0.05，**P<0.01，***P<0.001.

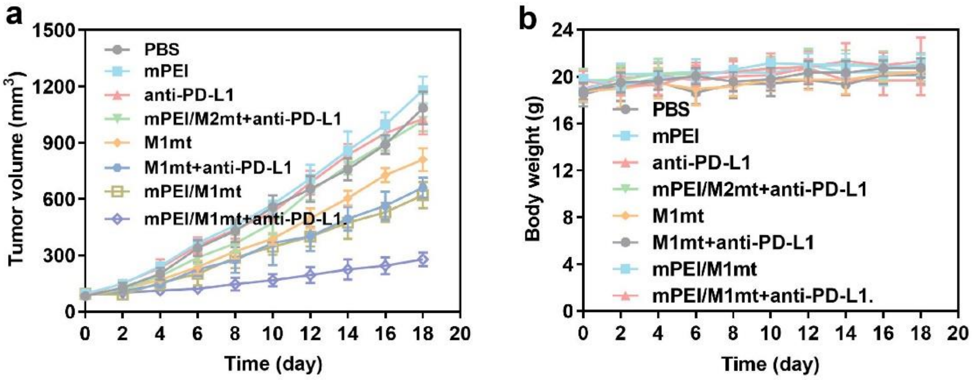

Figure S18. Tumor growth (a) and body weight (b) curves of 4T1 tumor-bearing mice under different treatments. Mean ± SD (n = 5).

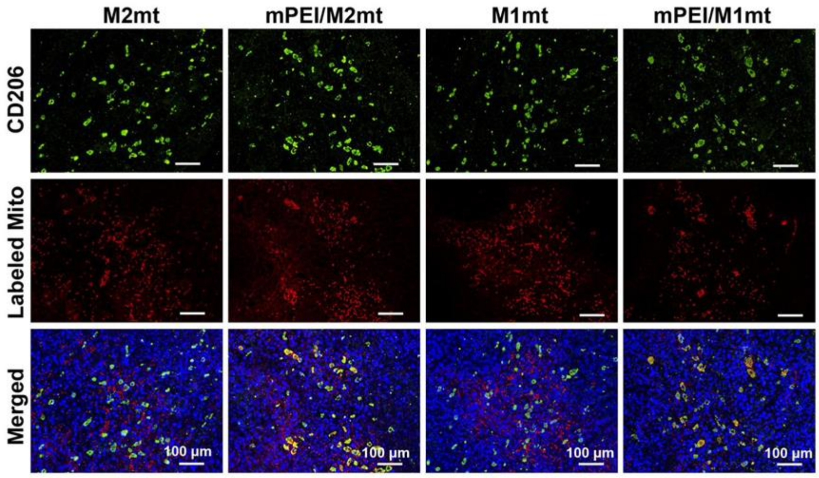

Figure S19. The localization of isolated mitochondria in tumors was observed using tumor tissue sections after 4h of injection. Red: isolated mitochondria; Green: CD206; Blue: nuclei.

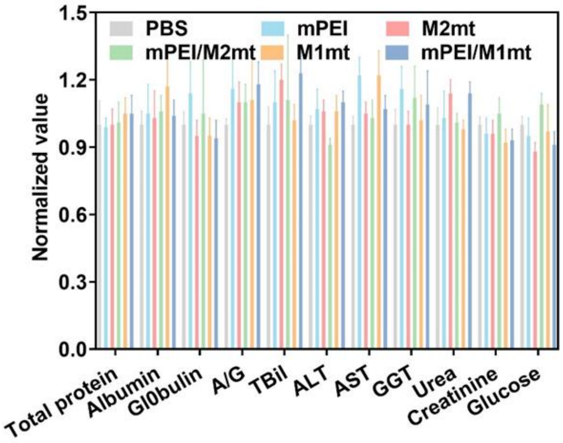

Figure S20. The comprehensive metabolic analysis of tumor-bearing mice with local injection of mPEI/M1mt and other agents. Peripheral blood was collected from the tail vein after 7 days of treatment and blood biochemical indexes were detected. Albumin/globulin (A/G), total bilirubin (TBil), alanine aminotransferase (ALT), aspartate aminotransferase (AST), γ-glutamyl transpeptidase (GGT), serum creatinine (Scr) and serum urea nitrogen (SUN). The error bar represents mean ± SD, n = 3.

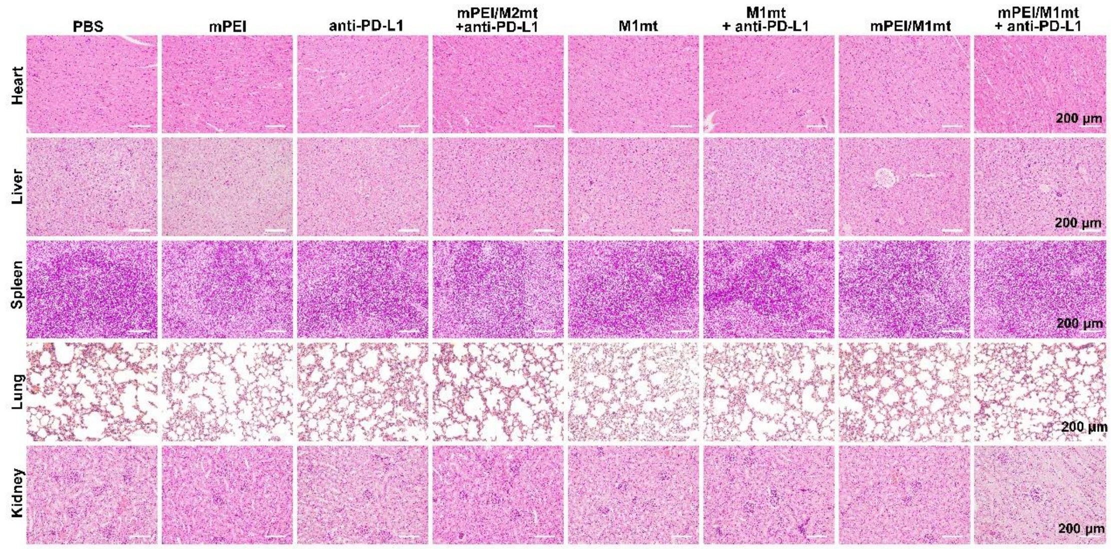

Figure S21. H&E staining of major organs collected from mice after different treatments of 7 days.

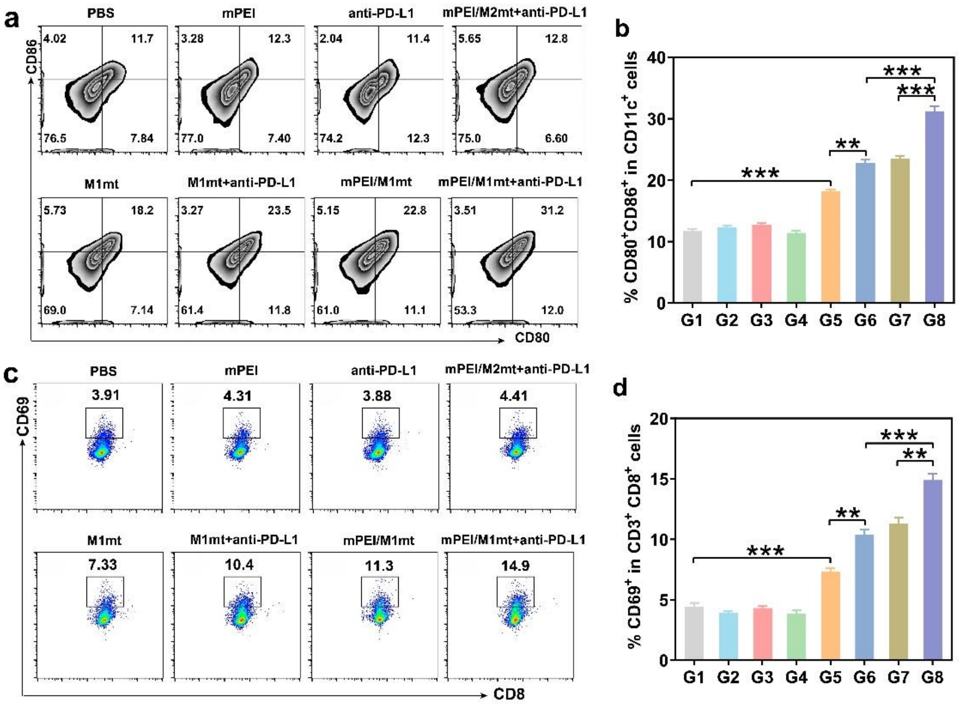

Figure S22. Evaluation of DC maturation and CD8 T cell activation in the 4T1 tumor-draining lymph node. (a-b) The FCM analysis and its corresponding statistical data of DC maturation (CD80$^+$ CD86$^+$ cells, gated by CD11c$^+$ cells) in tumor-draining lymph nodes after different treatments. (c-d) The FCM analysis and its corresponding statistical data of activated CD8 T cells (CD8$^+$ CD69$^+$ cells, gated by CD3$^+$ T cells) in tumor-draining lymph nodes after different treatments. G1: PBS, G2: mPEI, G3: anti-PD-L1, G4: mPEI/M2mt+anti-PD-L1, G5: M1mt, G6: M1mt+anti-PD-L1, G7: mPEI/M1mt, G8: mPEI/M1mt+anti-PD-L1. Mean±SD (n=3), *P<0.05, **P<0.01, ***P<0.001.

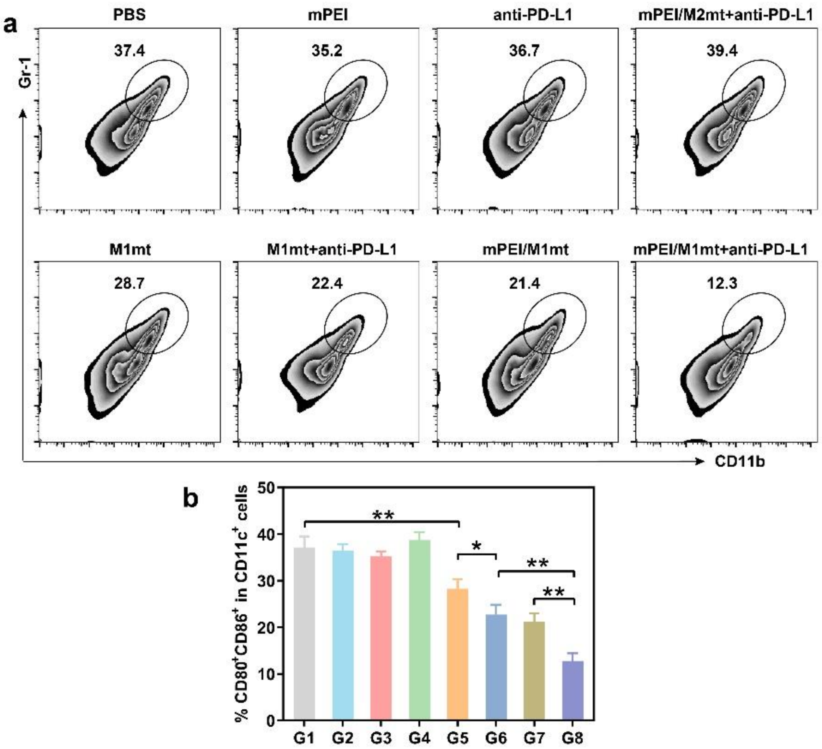

Figure S23. The FCM analysis and corresponding statistical data of MDSCs (CD45+ CD11b+ Gr-1+ cells, gated by CD45+ cells) in tumor tissues after different treatments. G1: PBS, G2: mPEI, G3: anti-PD-L1, G4: mPEI/M2mt+anti-PD-L1, G5: M1mt, G6: M1mt+anti-PD-L1, G7: mPEI/M1mt, G8: mPEI/M1mt+anti-PD-L1. Mean±SD (n=3), *P<0.05, **P<0.01, ***P<0.001.

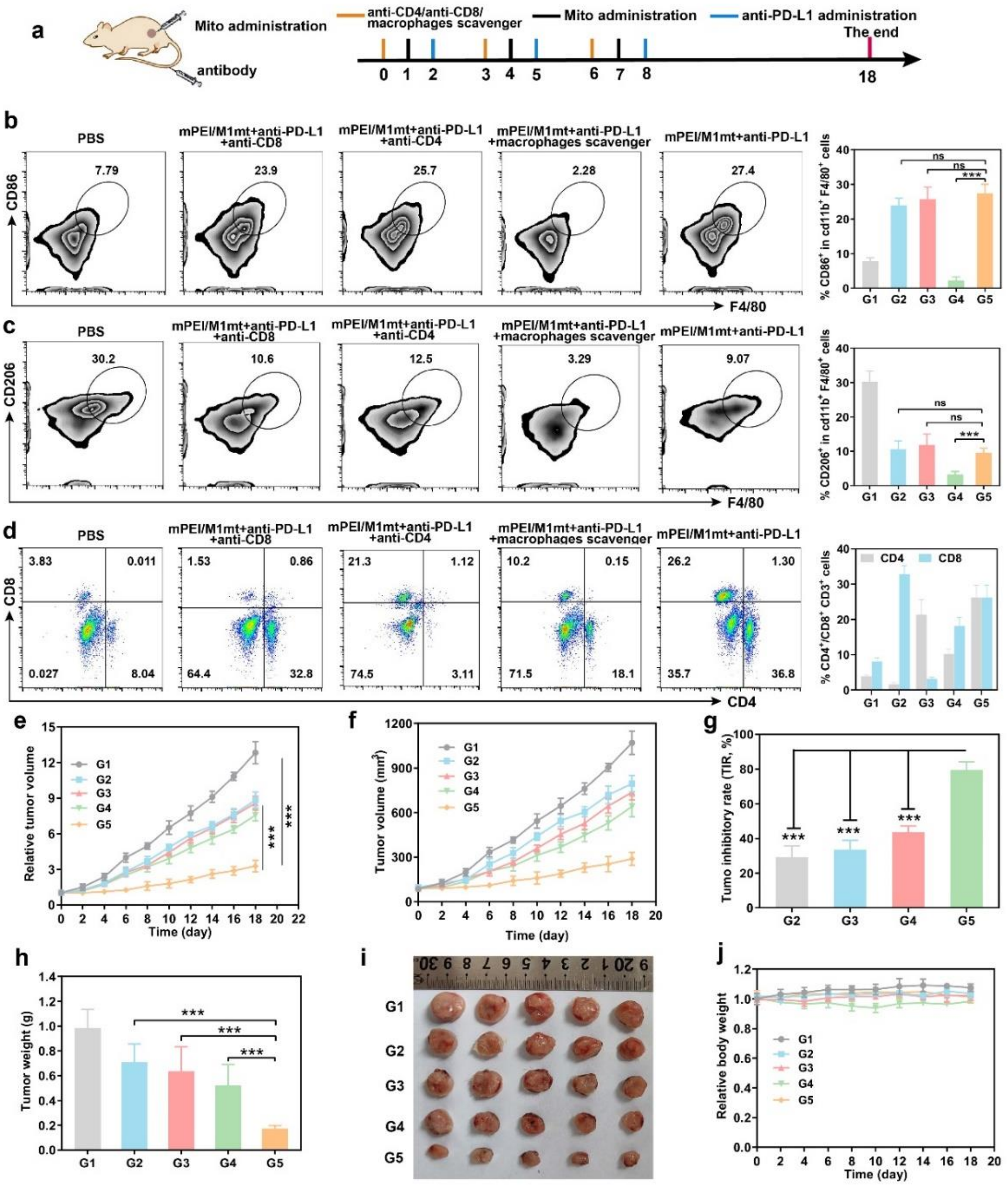

Figure S24. Antitumor effect of 4T1 tumor-bearing mice model in vivo after different cells depletion. (a) Schematic diagram of mouse administration procedures. (b-c) The FCM analysis of M1 macrophages (CD86+ F4/80+ cells) and M2 macrophages (CD206+ F4/80+ cells) in the tumor tissue after different treatments, cells are gated by CD11b+ cells, n=3. (d) The FCM analysis of $CD4^+$ T cells and $CD8^+$ T cells (gated by $CD3^+$ T cells) in tumor tissues after different treatments, n=3. (e-f) Relative and absolute tumor growth curves of tumor-bearing mice under different treatments. (g) Tumor inhibitory rate (TIR) of tumor growth. (h) Weight of the tumors harvested on day 18 after different treatments. (i) Digital images of tumors collected from mice on day 18 after different treatments. (j) The body weight curves changed over time in tumor-bearing mice under different treatments. G1: PBS, G2: mPEI/M1mt+anti-PD-L1+anti-CD8, G3: mPEI/M1mt+anti-PD-L1+anti-CD4, G4: mPEI/M1mt+anti-PD-L1+Macrophages scavenger, G5: mPEI/M1mt+anti-PD-L1. Mean±SD (n=5)，*P<0.05，**P<0.01，***P<0.001.

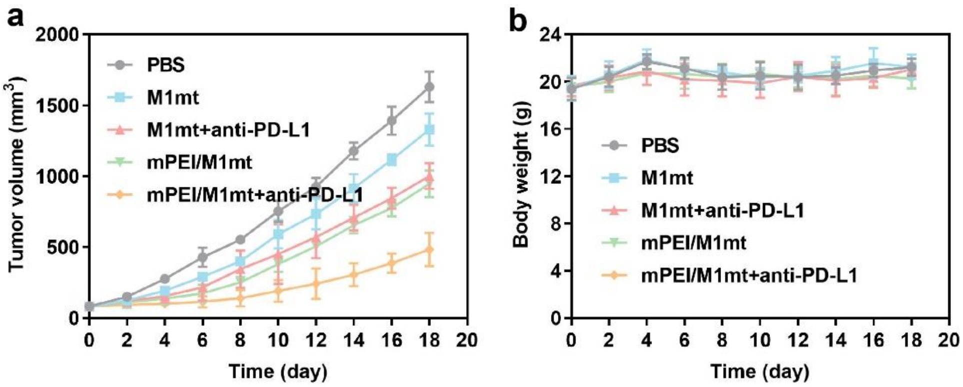

Figure S25. Tumor growth (a) and body weight (b) curves of CT26 tumor-bearing mice under different treatments. Mean ± SD (n = 5).

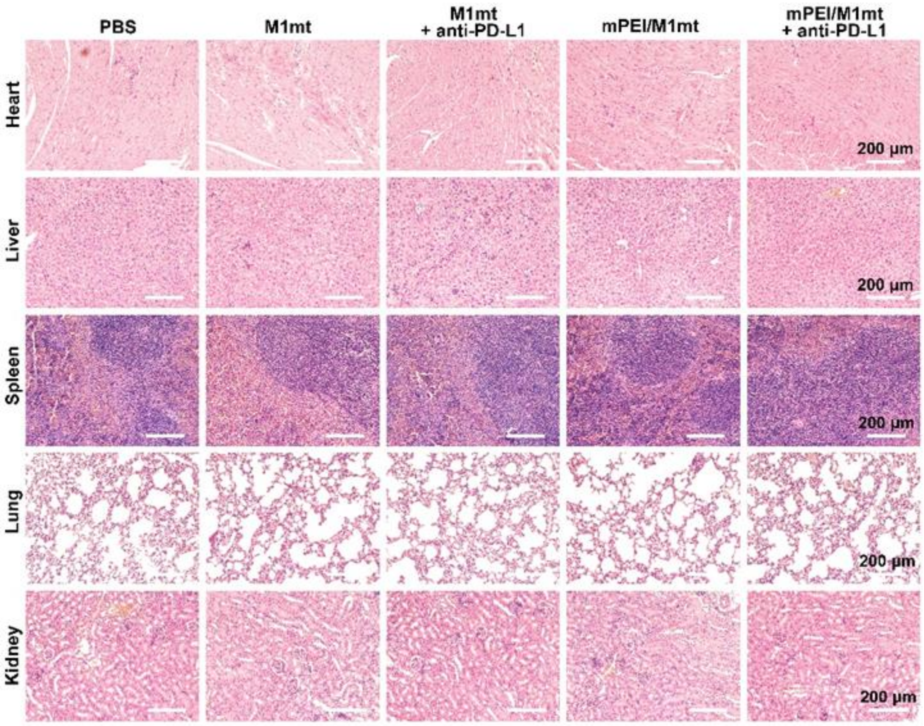

Figure S26. H&E staining of major organs collected from mice after different treatments of 7 days.

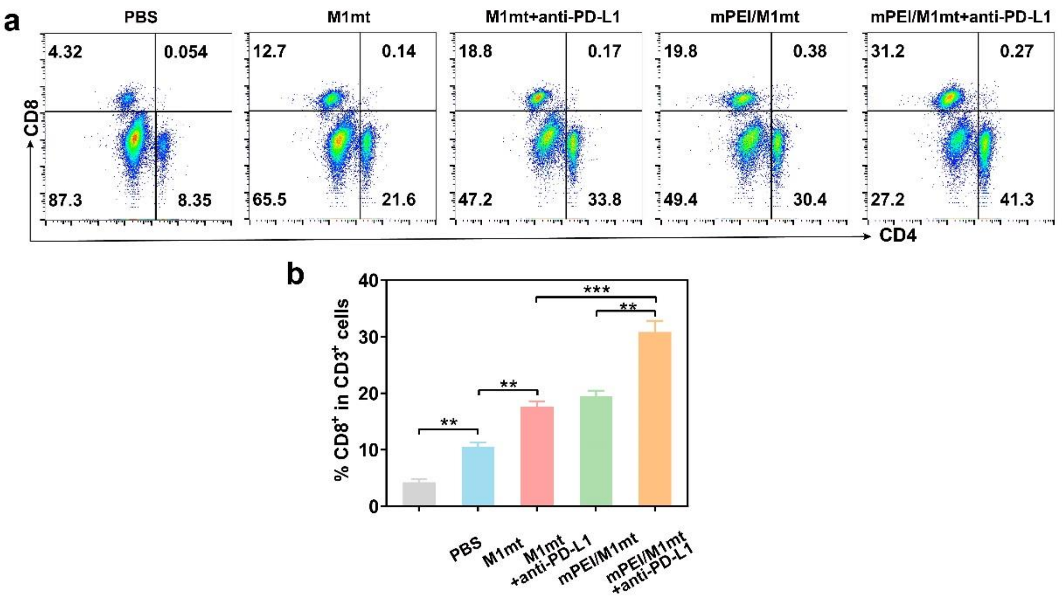

Figure S27. Immune response of mPEI-M1mt + anti-PD-L1 on CT26 cell-bearing mice. The FCM analysis and the corresponding quantitation of $CD8^+$ T cells in tumors after different treatments. Mean ± SD (n=3)，*P<0.05，**P<0.01，***P<0.001.
